# Tracking light-induced electron transfer toward O_2_ in a hybrid photoredox-laccase system

**DOI:** 10.1016/j.isci.2021.102378

**Published:** 2021-03-31

**Authors:** Rajaa Farran, Yasmina Mekmouche, Nhat Tam Vo, Christian Herrero, Annamaria Quaranta, Marie Sircoglou, Frédéric Banse, Pierre Rousselot-Pailley, A. Jalila Simaan, Ally Aukauloo, Thierry Tron, Winfried Leibl

**Affiliations:** 1 Université Paris-Saclay, CEA, CNRS, Institute for Integrative Biology of the Cell (I2BC), 91191 Gif-sur-Yvette, France; 2 Lebanese International University, 146404 Mazraa, Beirut, Lebanon; 3 Aix Marseille Université, Centrale Marseille, CNRS, iSm2 UMR 7313, 13397 Marseille, France; 4 Université Paris-Saclay, Institut de Chimie Moléculaire et des Matériaux d’Orsay (ICMMO), 91405 Orsay, France

**Keywords:** Chemistry, Catalysis, Biomolecules

## Abstract

Photobiocatalysis uses light to perform specific chemical transformations in a selective and efficient way. The intention is to couple a photoredox cycle with an enzyme performing multielectronic catalytic activities. Laccase, a robust multicopper oxidase, can be envisioned to use dioxygen as a clean electron sink when coupled to an oxidation photocatalyst. Here, we provide a detailed study of the coupling of a [Ru(bpy)_3_]^2+^ photosensitizer to laccase. We demonstrate that efficient laccase reduction requires an electron relay like methyl viologen. In the presence of dioxygen, electrons transiently stored in superoxide ions are scavenged by laccase to form water instead of H_2_O_2_. The net result is the photo accumulation of highly oxidizing [Ru(bpy)_3_]^3+^. This study provides ground for the use of laccase in tandem with a light-driven oxidative process and O_2_ as one-electron transfer relay and as four-electron substrate to be a sustainable final electron acceptor in a photocatalytic process.

## Highlights

• An electron relay boosts photoreduction of laccase
• Superoxide is efficiently captured by laccase preventing formation of H_2_O_2_
• Light activation reveals information on elementary steps inside the enzyme
• Laccase enables O_2_ as terminal electron acceptor for oxidative photocatalysis

## Graphical abstract

**Figure:**
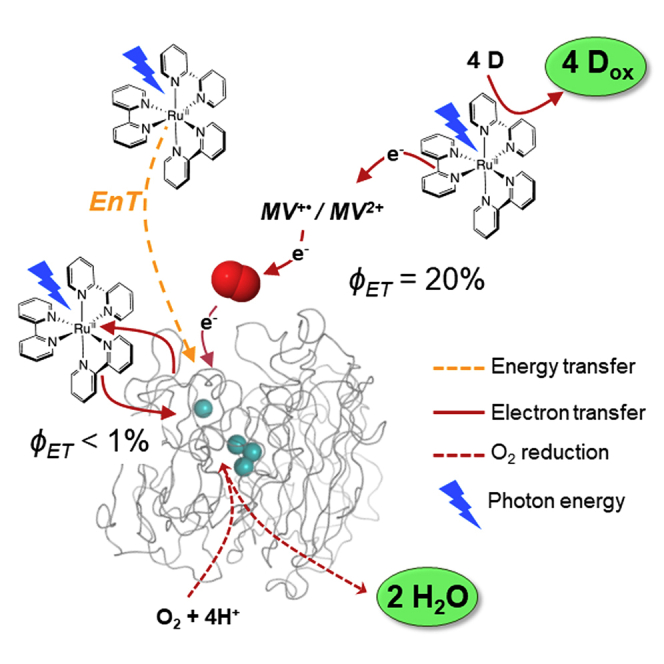

## Introduction

Electron transfer (ET) reactions are of fundamental importance in biological processes such as photosynthesis and respiration (Winkler, 2005). Understanding how electrons are transported in these complex systems may help chemists discover new tools to perform sustainable multielectronic chemical transformations. Currently, chemists and biologists are gathering their efforts to use light energy to drive enzymes to realize both chemical oxidation and reduction reactions of high importance for our societies (Lee et al., 2018; Litman et al., 2018; Guo et al., 2018; Schmermund et al., 2019). These targets are gaining much attention from both a fundamental and applied point of view due to the fact that to date we do not have catalytic systems that can match the activities and specificities of the biological catalysts. Hence, discoveries in this field may help develop sustainable chemical processes and also help in the design of more simple and robust photocatalytic systems (Pellegrin and Odobel, 2011).

Laccases are robust enzymes used in many industrial and biotechnological processes (Mate and Alcalde, 2017; Couto and Herrera, 2006; Agrawal et al., 2018; Riva, 2006). In nature, laccases couple the oxidation of organic substrates such as phenol derivatives, to the reduction of O_2_ to water (Madhavi and Lele, 2009; Tron, 2013). The catalytic mechanism of laccases has been extensively studied (Solomon et al., 2008; Jones and Solomon, 2015; Heppner et al., 2014). At a type 1 (T1) Cu ion site the one-electron oxidation of substrate molecules occurs, and electrons are sequentially transferred inside the protein to a trinuclear copper catalytic center (TNC) where O_2_ is eventually reduced in a four-electron, four-proton reaction (Jones and Solomon, 2015; Bertrand et al., 2002). The TNC consists of a pair of coupled type 3 (T3) Cu ions and a mononuclear type 2 (T2) Cu ion (Solomon et al., 1996; Hakulinen and Rouvinen, 2015). Although laccases are active on a wide range of natural substrates including phenol, polyphenols, hydroxyindoles, and benzenethiols among others, their oxidative power is locked by the one-electron oxidation process taking place at the T1 site whose potential ranges from 420 to 790 mV versus NHE, thus limiting the scope for the oxidation of organic substrates. Inspired by this biological tandem of oxidative and reductive processes performed by MCOs we have been interested in demonstrating that we can employ O_2_ as the final oxidant by coupling a photoredox cycle with a laccase. Light-induced ET from the excited state of a photosensitizer to laccase leads to the reduced enzyme and the generation of a highly oxidizing chromophore. In doing so, we can use the reductive facet of this family of enzymes as a substitute for the problematic and unsustainable sacrificial electron scavengers such as persulfate or cobalt(III) ions widely used in photocatalytic multielectronic oxidative processes (Herrero et al., 2011). From our first studies, it appears that the effective quantum yield for the reduction of the MCO using ruthenium(II) polypyridine-type chromophores in the presence of a sacrificial electron donor does not exceed 1% (Simaan et al., 2011). When a porphyrin-based chromophore is used, this yield approaches 35% (Lazarides et al., 2013) but suffers from an intrinsic low stability of the chromophore in the presence of O_2_. The poor efficiency observed with the ruthenium photosensitizers was initially conferred to the shorter excited state lifetime of [Ru(bpy)_3_]^2+^ (600 ns) as compared with the zinc porphyrin triplet state (720 μs). However, other factors such as energy transfer quenching and back electron transfer might also hamper this bimolecular light-activation process. Such pathways can hardly be assessed from measurements of overall yields under continuous illumination. To improve the efficiency of the light-driven activation of laccase by [Ru(bpy)_3_]^2+^, we set to reinvestigate the photophysical events occurring upon excitation of a [Ru(bpy)_3_]^2+^ chromophore and its interaction with laccase. In the present study we show that the efficiency of light-induced electron transfer between our synthetic photosensitizer and the laccase enzyme can be strongly enhanced, from 1% to 20%, by the introduction of methyl viologen (MV^2+^) as an electron relay. We track the role of methyl viologen acting as an efficient oxidative quencher of the photosensitizer excited triplet state to form a methyl viologen radical, MV^⋅+^, which efficiently reduces the laccase. We furthermore delineate the reaction pathways under aerobic conditions, where MV^⋅+^ reacts with O_2_ to form superoxide radicals that are efficiently scavenged by the enzyme to perform the 4-electron reduction of O_2_ to H_2_O. As a result of these processes the powerful and long-lived oxidant [Ru(bpy)_3_]^3+^ with an oxidizing power of *ca.* 1300 mV versus NHE is generated. At all stages experimental data are confronted with kinetic simulations to arrive at a consistent description of the reaction sequence and the interactions at play between the different components.

## Results and discussion

### Laccase photoreduction by [Ru(bpy)_3_]^2+∗^: Marcus versus Förster

We first reinvestigated the interaction between the [Ru(bpy)_3_]^2+^ photosensitizer and the laccase enzyme. Steady-state and time-resolved emission and absorption studies were performed on solutions containing [Ru(bpy)_3_]^2+^ and various concentrations of laccase (LAC3 from *Trametes* sp. C30) in de-aerated Britton-Robinson (B&R) buffer at pH 6. As illustrated in Figure S1 in the supplemental information, the [Ru(bpy)_3_]^2+^ chromophore is characterized by its 450-nm metal-to-ligand charge-transfer (MLCT) band (*ε* = 14,600 M^−1^ cm^−1^) (Kalyanasundaram, 1982a) with an emission band of its excited state at 610 nm, whereas the oxidized copper(II) ion at the T1 site of the laccase displays an absorption band at 610 nm (*ε* = 5600 M^−1^ cm^−1^) (Solomon et al., 2004). These spectroscopic probes were used accordingly to monitor the excited state of the photosensitizer and LAC3 photoreduction.

**Figure 1:**
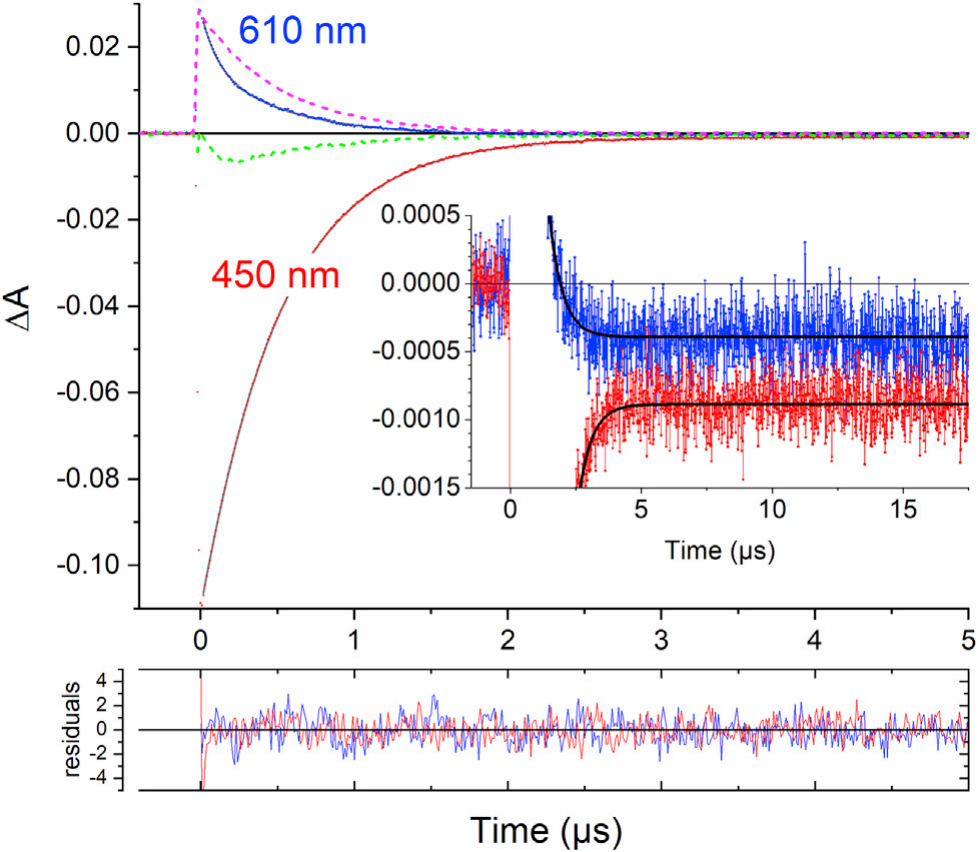
Laccase photoreduction by [Ru(bpy)_3_]^2+∗^ Transient absorption kinetics of a solution of [Ru(bpy)_3_]^2+^ (15 μM) and LAC3 (37 μM) in argon-purged B&R buffer, pH = 6 after laser flash excitation at 455 nm. Inset: vertical zoom of the data. The absorption transient at 450 nm (red trace) indicates formation of 9.7 μM of [Ru(bpy)_3_]^2+∗^ by the nanosecond laser flash decaying to a residual long-lived negative absorption difference of 0.8 × 10^−3^ indicating formation of 0.063 μM of [Ru(bpy)_3_]^3+^ and thus a yield of 0.65% for photoinduced ET. The bleaching observed at 610 nm at long times (blue trace, Δ*A* = −0.0004) indicates reduction of 0.07 μM of T1 in good agreement with the quantity of oxidized chromophore. Used extinction coefficients: Δ*ε*Ru^∗^_450_ = *ε*Ru^∗^_450_ – *ε*Ru^2+^_450_ = −11,300 M^−1^ cm^−1^ (Muller and Brettel, 2012); Δ*ε*Ru^3+^_450_ = *ε*Ru^3+^_450_ – *ε*Ru^2+^_450_ = −12,600 M^−1^ cm^−1^ (Kalyanasundaram, 1982b); *ε*T1_610_*=* 5600 M^−1^ cm^−1^ (Solomon et al., 2004). Small contributions to absorption changes at 450 nm due to T1 reduction and at 610 nm due to Ru^3+^ were neglected. The dashed pink line is the scaled kinetics of emission at 610 nm, indicating that the 610 nm absorption transient contains contribution from other species than the excited state. Green dashed line: difference between the dashed pink line and the 610 nm emission kinetics representing transient bleaching of T1 absorption. Bottom: residuals for best fit according to the reaction scheme described in the text (Scheme 1) and in the supplemental information. Best fit traces are superimposed on the red and blue experimental traces as black lines both in the main window and in the inset.

Time-resolved emission measurements with increasing LAC3 concentrations indicate a dynamic quenching process of the ruthenium excited state by laccase with a diffusion-limited bimolecular rate constant of 6.1 10^9^·M^−1^s^−1^ (Figure S2). This emission quenching could be due to energy and/or electron transfer from the sensitizer to the laccase. If all the quenching were due to ET a quantum yield of 12% of laccase reduction (compared with the concentration of excited state formed by the laser excitation) would be expected for a concentration of laccase of 37 μM. However, the amplitude of the measured absorption changes at long times, i.e., after decay of the excited state (Figure 1, inset) indicates a yield for the laser-flash-induced formation of the charge-separated state (CSS) of [Ru(bpy)_3_]^3+^ and reduced LAC3 (Cu^I^) of only 0.7% (see Figure 1 for details). This yield is 17 times lower than what is expected for the products of the quenching process, suggesting that less than 10% of the quenching reactions lead to an effective reduction of laccase despite the high driving force for the ET from the ruthenium to the copper center T1 of –Δ*G* = 1.52 eV estimated from the redox potentials (*E*(Ru^III^/Ru^II∗^) = −0.84 V; *E*(Cu^II^/Cu^I^) = 0.68 V) (Kalyanasundaram, 1982a; Balland et al., 2008; Bock et al., 1979). These findings suggest that the major part of quenching is related to a Förster-type energy transfer process that can be expected to be rather efficient due to the strong spectral overlap between the [Ru(bpy)_3_]^2+^^∗^ emission spectrum and the absorption band of the Cu^II^ of the T1 site (Figure S1) (Winkler, 2013). The Förster radius (*R*_0_ = 2.6 nm) calculated for this interaction implies that upon encounter, through-space resonance energy transfer will largely outcompete ET, the rate of which is seven orders of magnitude slower at that distance (see supplemental information, transparent methods Section and Figure S5).

Interestingly, a careful comparison of the kinetics of the emission transient at 610 nm, expected to represent the decay of the [Ru(bpy)_3_]^2+^^∗^ excited state, and the absorption transient at 610 nm reveals significant differences in the kinetics (Figure 1, compare blue and dashed pink lines). It appears clearly that the transient absorption trace recorded at 610 nm contains, in addition to the positive absorption attributable to the [Ru(bpy)_3_]^2+^^∗^ excited state that has a small absorption at this wavelength (Yoshimura et al., 1993), a transient bleaching that can be related to a loss of Cu^2+^ absorption of the T1 site. The transient bleaching at 610 nm (green dashed line in Figure 1) is well described by two exponential functions for its formation and decay with rate constants of 10^7^ s^−1^ and 2 × 10^6^ s^−1^, respectively. Considering a concentration of laccase of 37 μM, the observed forward rate is too fast to be compatible with a diffusion-limited reaction between the sensitizer and the laccase. This fact leads us to postulate the formation of a relatively strong association complex between the [Ru(bpy)_3_]^2+^ chromophore and LAC3 (*K*_a_ = 4.75 mM^−1^; determined by kinetic simulations described below), present in equilibrium with the separated species before and directly after the excitation flash. Such association complexes between ruthenium polypyridine and other transition metal complexes with blue copper proteins have been reported before (Brunschwig et al., 1985; Goldberg and Pecht, 1976). This interaction is usually attributed to the presence of negatively charged regions on the protein surface interacting with the positively charged metal complexes. The crystal structure of a laccase from *Trametes versicolor* revealed a dominance of negative charges of the protein and the substrate binding site as a small negatively charged cavity near copper T1 (Piontek et al., 2002). However, interactions other than electrostatic may occur (Kurzeev et al., 2009). For LAC3, molecular docking calculations predict a proximal binding position for [Ru(bpy)_3_]^2+^ at a distance as short as 11.2 Å from the Cu T1 (Robert et al., 2017). We note that the association constant *K*_a_ = 4.75 mM^−1^ implies that under the conditions of the experiment shown in Figure 1, 12.5% of the [Ru(bpy)_3_]^2+^ complexes (1.9 μM of 15 μM employed) are in association with a LAC3 protein.

**Scheme 1:**
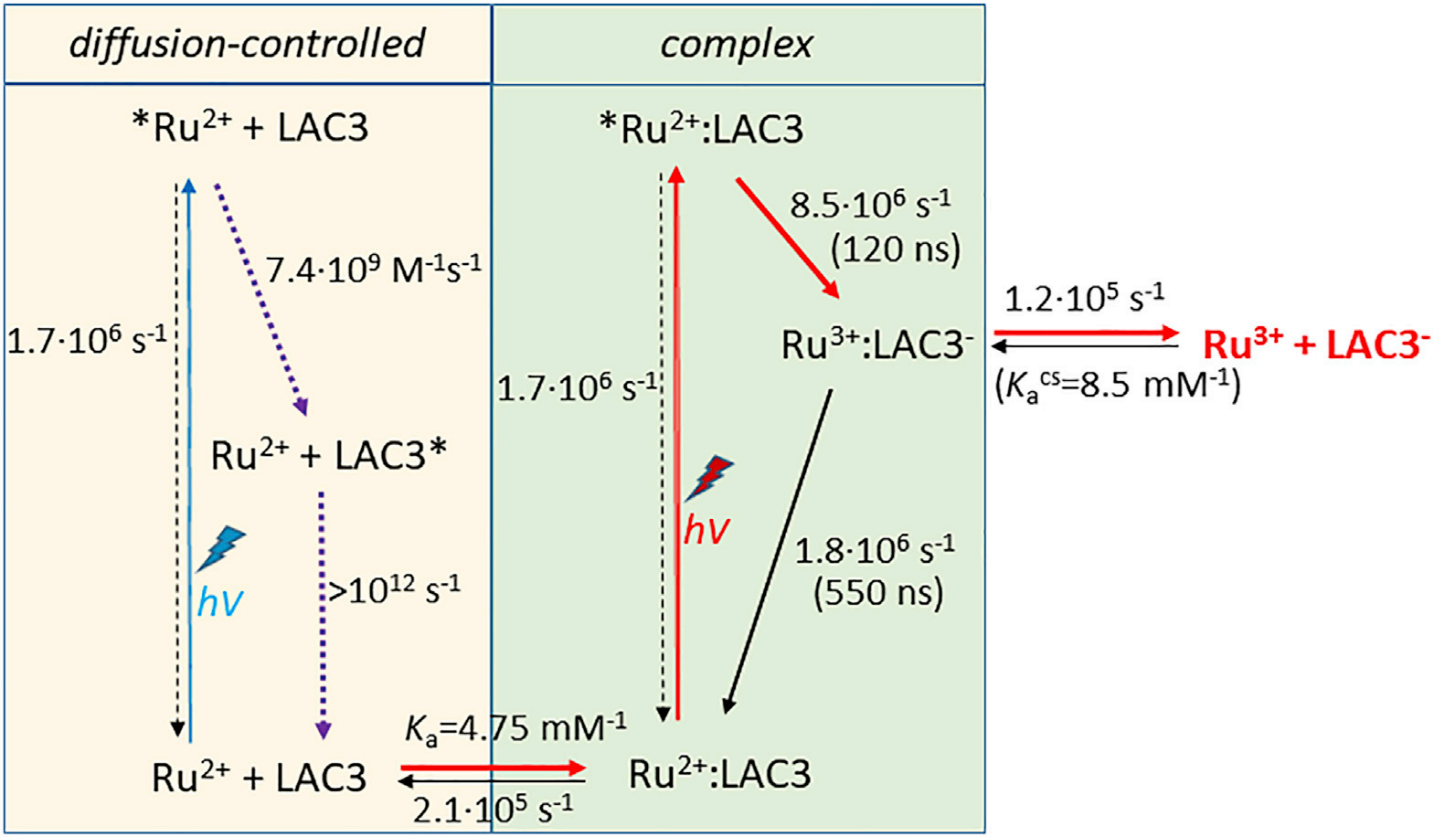
Interactions between the [Ru(bpy)_3_]^2+^ photosensitizer and the LAC3 enzyme as identified by global analysis of laser flash photolysis experiments Reactions leading to formation of reduced laccase (LAC3^-^) and oxidized photosensitizer (Ru^3+^) are indicated by red arrows. In the bimolecular pathway (left) Förster resonance energy transfer quenching of the [Ru(bpy)_3_]^2+^^∗^ excited state largely outcompetes ET to the T1 Cu^II^ center in LAC3. In the association complex (right) charge recombination is about 10 times faster than dissociation leading to a low yield for formation of the target state Ru^3+^ LAC3^–^ (red). Parameter values were deduced from analysis of the data of Figure 1 (pH 6).

The question that arises then is whether the observed bleaching of T1 absorption in the association complex should be attributed to an electron or energy transfer process from the [Ru(bpy)_3_]^2+^ excited state to the copper T1. We exclude formation of the doublet excited state of the T1 copper ion, as this state was shown to decay to the ground state in less than 1 ps, which would certainly not allow us to observe a bleaching of T1 absorption persisting for hundreds of ns (Delfino et al., 2015). Formation of another, more long-lived excited state of the T1 Cu ion via Förster resonance energy transfer or via simultaneous exchange of an electron between T1 and the excited [Ru(bpy)_3_]^2+^ complex (Dexter mechanism) could explain the transient bleaching of T1 absorption. However, both energy transfer processes should lead to a concomitant recovery of the [Ru(bpy)_3_]^2+^ ground state for which no indication is detectable in the transient absorption trace recorded at 450 nm. For this reason and to account for the formation of stable Ru^3+^ and reduced T1 (Figure 1) we attribute the observed transient bleaching of T1 to a photoinduced electron transfer reaction according to (Equation A):

(Equation A) $${}^{*}Ru^{2+}:T1\overset{k_{cs}}{\to }\;Ru^{3+}:T1^{–}\;\overset{k_{br}}{\to }\;Ru^{2+}:T1$$

where the rate constant *k*_cs_ describes charge separation from the excited state of the chromophore yielding the oxidized state of the chromophore (Ru^3+^) and reduced laccase (T1^–^), and *k*_br_ describes charge recombination within the complex. It should be noted that the transiently formed species Ru^3+^ is characterized by an extinction coefficient close to the one of the ^∗^Ru^2+^ excited state (see legend of Figure 1) that leads to only very weak modifications of the absorption at 450 nm. The dataset of Figure 1 was analyzed by a global kinetic simulation based on the complete reaction scheme (Scheme 1, see also transparent methods section in the supplemental information), and an excellent fit to the data was obtained (solid black traces and residuals in Figure 1) with all rate constants well defined. From the *k*_on_ and *k*_off_ values describing formation of the association complex at two different concentrations of LAC3 an association constant of *K*_a_ = 4.75 mM^−1^ was determined together with values for the rate constants *k*_cs_ and *k*_br_, of 8.5 × 10^6^ s^−1^ and 1.8 × 10^6^ s^−1^, respectively. Considering the large difference in driving forces for the charge separation and charge recombination reactions (–Δ*G*^*cs*^ = 1.52 eV versus –Δ*G*^*br*^ = 0.58 eV), the rate constants are surprisingly close. To obtain more information on the driving force dependance of these ET rates the same process was investigated using [Ru(bpz)_3_]^2+^ (bpz: bipyrazine), a ruthenium sensitizer with significantly higher Ru^3+^/Ru^2+∗^ and Ru^3+^/Ru^2+^ potentials (0.13 V and +2.23 V for [Ru(bpz)_3_]^2+^ compared with −0.84 V and +1.26 V/NHE for [Ru(bpy)_3_]^2+^ (Kalyanasundaram, 1982b; Wehlin et al., 2017). Consequently, with the [Ru(bpz)_3_]^2+^ sensitizer the driving force for charge separation is about 1 eV lower than with [Ru(bpy)_3_]^2+^, whereas the driving force for charge recombination is increased by the same amount. Global kinetic simulation (Figure S3) revealed an affinity of [Ru(bpz)_3_]^2+^ for LAC3 about 2 times lower than in the case of [Ru(bpy)_3_]^2+^ (*K*_a_ = 2.36 mM^−1^ versus 4.75 mM^−1^), implying a lower steady-state concentration of complexes in the case of [Ru(bpz)_3_]^2+^. The rate constants for the two ET reactions in the association complex between [Ru(bpz)_3_]^2+^ and LAC3 were determined as *k*_cs_ = 6.6 × 10^6^ s^−1^and *k*_br =_ 1.2 × 10^6^ s^−1^, close to the values found in the case of [Ru(bpy)_3_]^2+^.

With the known driving forces for the two reactions in eq (A) for the two sensitizers, the rates could be analyzed in the framework of Marcus theory permitting an estimation of the reorganization energy and distances for the ET reactions (Figure S4; see also transparent methods Section in the supplemental information). A value of 1.05 ± 0.07 eV for the reorganization energy of ET for both charge separation and recombination can be deduced, which places the two ET reactions with high driving force in the inverted region of the Marcus curve (Figure S4). The value for the reorganization energy is consistent with values reported in literature for oxidation or reduction of T1 copper sites (0.6–0.8 eV) and the low internal reorganization energy of the Ru^2+^/Ru^3+^ (or Ru^2+∗^/Ru^3+^) transition (Di Bilio et al., 1997; Farver et al., 2004; Wijma et al., 2007; Mines et al., 1996). Also, the distances extracted from the Marcus analysis, *r* = 12.5–13.5 Å, compare reasonably well with the Ru^II^-to-T1 Cu^II^ distance of 11.2 Å found by non-covalent docking simulations (Robert et al., 2017). Interestingly, when the rates for charge separation and recombination were analyzed separately (red and blue fit curves in Figure S4) the donor-acceptor distance for charge recombination was found to be 1 Å larger than the distance for charge separation. Clearly additional data points on the Marcus plot would be desirable to draw definite conclusions, but it is tempting to attribute this difference to the location of the electron transferred upon charge separation on the peripheral bipyridine ligand in the ^∗^Ru^2+^ excited state in contrast to the location of the positive charge receiving the electron during back ET on the central metal ion Ru^3+^. The fact that all rates determined by our global simulation can be consistently described by Marcus theory with meaningful parameters for distances and reorganization energy further supports the proposed mechanism of electron transfer described by eq. (A).

The flash-induced energy and electron transfer reactions occurring upon excitation of [Ru(bpy)_3_]^2+^ in a solution containing LAC3 are summarized in Scheme 1 and values for the case of [Ru(bpz)_3_]^2+^ are shown in Scheme S1 in the supplemental information. It can be concluded from this investigation that diffusion-controlled electron transfer from [Ru(bpy)_3_]^2+^^∗^ to LAC3 is short circuited by Förster energy transfer because the latter operates over longer distances and that only excitation of a sensitizer present in a Ru:LAC3 association complex at the time of photon absorption leads to electron transfer to the enzyme. The right-side mechanism in Scheme 1 further predicts that the yield of effective laccase reduction is given by the competition between the dissociation of the [Ru(bpy)_3_]^3+^:LAC3^–^ complex described by the rate *k*_off_ and charge recombination within the complex described by the rate *k*_br_. The association constant for the charge separated state can be determined from the value of *k*_off_ resulting from the global fit (*k*_off_ = 1.2 × 10^5^ s^−1^), yielding *K*_a_^cs^ = 8.5 mM^−1^. The slightly increased affinity of the sensitizer for LAC3 in the charge separated state (*K*_a_^cs^ = 8.5 mM^−1^ versus *K*_a_ = 4.75 mM^−1^) can be attributed to an increased electrostatic interaction between oxidized Ru^3+^ and reduced laccase. The ratio of *k*_off_/(*k*_off_ + *k*_br_) then gives a yield of 6% for the effective formation of Ru^3+^ and LAC3^–^ from the charge separated state. With only 12.5% of the ruthenium sensitizers engaged in such a complex, the overall quantum yield of persistent, not only transient, laccase reduction (and Ru^3+^ formation) drops to 0.75%. Put together, these different phenomena explain well the observed low yield of photoreduction of laccase from the excited state of the ruthenium sensitizer.

Although the simple reaction sequence proposed to occur in the association complexes (right panel of Scheme 1) describes well the spectroscopic data, it is surprising that no energy transfer reaction from [Ru(bpy)_3_]^2+^^∗^ to LAC3 had to be considered in the analysis. In other words, it appears that in the complex the rate of energy transfer is slower than the rate of ET. This situation is reminiscent of a recent observation in a flexible BODIPY-C_60_ dyad where photoinduced ET occurs in the folded conformer (*R* = 8.8 Å), whereas energy transfer is observed in extended conformers (*R* = 17–20 Å) (Tran et al., 2020). Similarly, in studies on single-walled carbon nanotubes tethered with porphyrins, excited-state energy transfer was found to be absent in the sample with a short tether (Li et al., 2004). Our results therefore seem not to be a unique case where at close distance the rate of energy transfer appears much slower than expected from the classical Förster rate-distance dependence. Although the different distance dependences between Förster energy transfer and ET (*R*^−6^ versus exponential) is expected to lead to a crossing of the *k*(*r*) curves with the rate of electron transfer becoming faster than the rate of energy transfer for short distances, in our case this crossing occurs at too short distance to provide a simple explanation for the dominance of ET over energy transfer (Figure S5). It might be argued that Förster energy transfer theory is an approximation based on point dipole-dipole interaction restricted to weak coupling, valid at distances greater than *R*_0_ (dashed green line in Figure S5), and extrapolation to significantly shorter distances might have to be taken with care (Braslavsky et al., 2008; Főrster, 1959). However, there exist a huge body of experimental data where the theoretical predictions hold even for quit short distances (Stryer and Haugland, 1967). We therefore favor another explication related to the spin multiplicity in our system with a ^∗^Ru^2+^ triplet state and a doublet ground state of the copper T1. Although examples of triplet-singlet and triplet-doublet energy transfer reactions have been described (Naqvi, 1981; Cravcenco et al., 2019), the selection rules for angular momentum conservation might result in a strong decrease of the energy transfer rates. A particularly striking example is the study by McCusker and coworkers on a model system where energy transfer was occurring or inhibited as a function of the spin state of the acceptor (Guo et al., 2011). In our system, even two orders of magnitude decrease in energy transfer rate due to the doublet spin state of the T1 Cu(II) could have little effect at long distances (≈*R*_0_) where the rate of electron transfer is slow but could be crucial at short distances where the rate of electron transfer is fast enough to compete (Figure S5). It would be interesting to investigate on the predominance of energy versus electron transfer in covalently linked ruthenium-laccase constructs where the distance can be varied in a controlled manner.

Considering the results described above it appears clearly that an efficient reduction of laccase cannot be obtained directly from the excited state of the sensitizer. In the following we assessed the possibility to overcome these limitations by using methyl viologen (MV^2+^) as an electron relay between [Ru(bpy)_3_]^2+^^∗^ and LAC3. Systems combining [Ru(bpy)_3_]^2+^ and MV^2+^ are probably the most extensively studied photoredox systems (Bock et al., 1974; Sun et al., 1994; Mandal and Hoffman, 1984; Wilson et al., 1998). Acting as an oxidative quencher to the photosensitizer's excited state, this well-known mediator is converted into a long-lived reduced species that could deliver electrons extracted from the chromophore to the enzyme even when the latter is present at low concentration. Such an approach has helped to convey electrons to redox enzymes such as hydrogenases under anaerobic conditions (Noji et al., 2014; Honda et al., 2016; Okura et al., 1979).

### Laccase photoreduction via MV^⋅+^

To act as mediator, the chemical species to be used must meet several requirements. Besides a suitable redox potential, it should have good solubility in aqueous solution, the oxidized form (necessarily present in mM concentration for good dynamic quenching of the photosensitizer's excited state) should have little absorption in the visible to avoid absorption of excitation light, and, most importantly, the reduced molecule must be able to act as a reductant for the enzyme. In contrast to many other small redox molecules investigated as mediator, MV^2+^ was found fit for this duty. It offers the additional advantage that the characteristic absorption features of its reduced state at 395 nm and 605 nm permit an easy tracking of its reduction and reoxidation. Under anaerobic conditions, the emission of [Ru(bpy)_3_]^2+^^∗^ was quenched by the addition of 10 mM MV^2+^ with a shortening of the lifetime of the excited state from 600 ns to 110 ns. The classic oxidative quenching of the [Ru(bpy)_3_]^2+^^∗^ by MV^2+^ involves a diffusion-limited encounter followed by an intermolecular electron transfer process (Kalyanasundaram, 1982c; Sun et al., 1994). The charge separated state (CSS) [Ru(bpy)_3_]^3+^ - MV^⋅+^ formed by dissociation of the encounter complex in competition with the back ET is described by a cage escape yield of about 20% (Sun et al., 1994). The addition of LAC3 (17 μM) to the [Ru(bpy)_3_]^2+^/MV^2+^ mixture did not result in a significant change of the [Ru(bpy)_3_]^2+^^∗^ excited state lifetime. This experimental observation suggests that the dominating deactivation pathway of the excited triplet state [Ru(bpy)_3_]^2+^^∗^ in the presence of both MV^2+^ and LAC3 is an ET process to MV^2+^, which is faster than energy transfer to the laccase because of the much higher concentration of MV^2+^ compared with LAC3. Transient absorption spectra were recorded at different time delays after the laser flash (Figure S6). At 1 μs after the laser pulse, a bleaching around 450 nm and the typical positive absorption features of the MV^⋅+^ radical (395 nm and 605 nm) were observed indicating the formation of about 1 μM of the [Ru(bpy)_3_]^3+^ - MV^⋅+^ CSS by the oxidative quenching mechanism discussed above. Spectral evolution up to 500 μs shows that the signature of reduced methyl viologen disappears completely followed by a bleaching around 610 nm, which denotes the consumption of the MV^⋅+^ radical and the loss of oxidized T1 in LAC3. These findings can be assigned to the charge shift from MV^⋅+^ to the T1 locus of the laccase while the bleaching due to the [Ru(bpy)_3_]^3+^ species partially persists (Figure S6, red trace).

**Figure 2:**
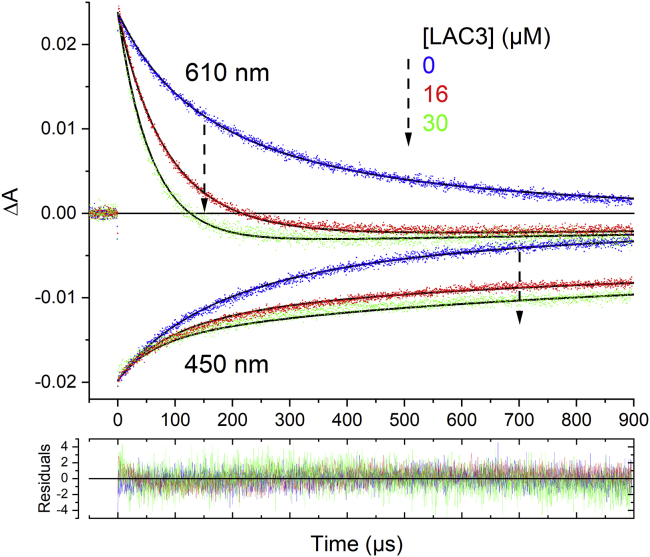
Laccase photoreduction via MV^⋅+^ Kinetic traces of a mixture of [Ru(bpy)_3_]^2+^ (25 μM) and MV^2+^ (10 mM) in B&R buffer, pH = 4, with different concentrations (0 μM, 16 μM, 30 μM) of LAC3. Absorption transients were recorded at 610 nm (upper part) or 450 nm (lower part). Black lines show the results of a global fit yielding values for the bimolecular rate constants for charge recombination between MV^⋅+^ and Ru^3+^ (3.1 × 10^9^ M^−1^s^−1^), electron transfer from MV^⋅+^ to T1 (0.38 × 10^9^ M^−1^s^−−1^), and oxidation of reduced T1 by Ru^3+^ (charge recombination, 0.46 × 10^9^ M^−1^s^−1^). Performing the experiment at pH 6 gave comparable results.

The absorption transients at specific wavelengths (Figure 2) allowed us to follow the concentration of the different intermediate species. In the [Ru(bpy)_3_]^2+^/MV^2+^/LAC3 system, excitation of [Ru(bpy)_3_]^2+^ leads to the generation of 7.8 μM [Ru(bpy)_3_]^2+∗^ triplet excited state as deduced from the maximum bleaching observed at 450 nm immediately after the laser flash (see legend of Figure 1 for extinction coefficients used). As described before, the excited [Ru(bpy)_3_]^2+^^∗^ transfers an electron to the MV^2+^ leading to the formation of 1.52 μM [Ru(bpy)_3_]^3+^ detected by the absorption bleach at 450 nm after decay of the excited state, and the same amount of MV^⋅+^ radical, observed by the absorption increase at 610 nm at early times after the flash (*ε*MV^+⋅^_605_ = 13700 M^−1^ cm^−1^) (Watanabe and Honda, 1982). From these numbers a quantum yield of *ca*. 19.5% for formation of the MV^⋅+^ radical is estimated. This yield agrees with reported values for the cage escape yield for reduced MV^⋅+^ from the encounter complex (Kalyanasundaram, 1982c; Sun et al., 1994). In the absence of LAC3 the MV^⋅+^ radical disappears in a second-order reaction (*k* = 3.1 × 10^9^ M^−1^ s^−1^) due to charge recombination with [Ru(bpy)_3_]^3+^ yielding mirror-like absorption transients at 450 and 610 nm (Figure 2 blue traces). In the presence of 16 μM LAC3 (red traces) the decay of MV^⋅+^ at 610 nm is strongly accelerated, and the absorption transient turns into a bleaching indicating loss of oxidized T1 of the laccase. With higher concentration of LAC3 the decay of MV^⋅+^ is further accelerated (Figure 2, green traces). The recovery of [Ru(bpy)_3_]^2+^ (bleaching at 450 nm) on the other hand slows down in the presence of laccase, and the lifetime of both species, reduced T1 and oxidized chromophore, extends into the ms time range. Using the extinction coefficients at 610 nm *ε*T1_610_ ≈ 5600 M^−1^ cm^−1^ and *ε*Ru^3+^_610_ ≈ 2000 M^−1^ cm^−1^ (Kalyanasundaram, 1982b), a concentration of 0.7 and 0.9 μM of reduced T1 was estimated to be formed after the laser flash for the two laccase concentrations, which represents a yield of 46 and 59% compared with the quantity of MV^⋅+^ produced. This clearly illustrates that MV^⋅+^ can reduce the laccase (Δ*G* = −1.13 eV) with diffusion-limited kinetics (*k*_2_ = 3.8 × 10^8^ M^−1^ s^−1^). Importantly, the quantum yield of formation of [Ru(bpy)_3_]^3+^ and [Cu^I^] depends only on the competition between the forward ET from MV^⋅+^ to LAC3 and the charge recombination of the [Ru(bpy)_3_]^3+^ - MV^⋅+^ CSS. This competition is in favor of laccase reduction even for low concentrations of LAC3 because the flash-induced concentration of MV^⋅+^ (and [Ru(bpy)_3_]^3+^, around 1 μM) is much smaller than the LAC3 concentration. It is important to recall that in this system, optimization of laccase reduction is equivalent to optimization of formation of the oxidized form of the ruthenium chromophore. The energetics and kinetics of the reactions occurring in the [Ru(bpy)_3_]^2+^/MV^2+^/LAC3 system are summarized in Scheme S2 in the supplemental information.

### Laccase photoreduction under continuous illumination in absence of O_2_

Time-resolved laser flash photolysis studies such as those presented above are an important tool to investigate the sequence of light-induced reaction steps and determine the relevant rate constants. On the other hand, photocatalytic processes employ continuous irradiation where reactions are governed by steady state concentrations significantly different from those encountered in synchronized flash excitation studies. To study the role of the electron mediator between the chromophore and LAC3 in the photoreduction process under continuous illumination, EDTA was added as an electron donor to the [Ru(bpy)_3_]^2+^/MV^2+^/LAC3 mixture. The [Ru(bpy)_3_]^3+^ state is the only species able to oxidize EDTA, regenerating the light-active [Ru(bpy)_3_]^2+^ state that can engage in subsequent ET reactions until the full four-electron reduction of laccase is achieved.

First, we studied LAC3 photoreduction in the absence of O_2_. In this context it is important to recall that under anaerobic conditions the fully oxidized form of the enzyme corresponds to the so-called “resting oxidized” (RO) form, which is structurally and functionally different from the fully oxidized native intermediate (NI) produced during catalytic turnover in the presence of O_2_. RO is proposed to evolve from NI by protonation keeping all four Cu centers oxidized but ambiguity concerning the details of reduction of the Cu centers in RO exists (Solomon et al., 2008; Jones and Solomon, 2015; Heppner et al., 2013, 2014).

**Figure 3:**
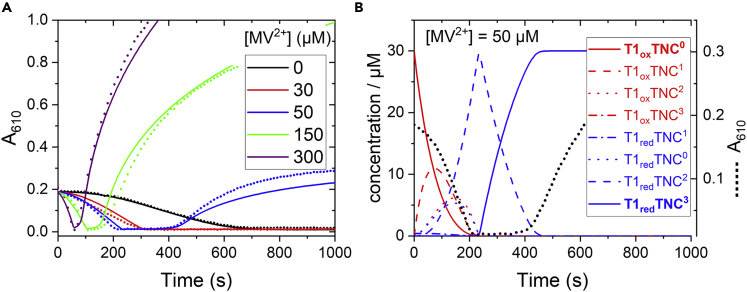
Laccase photoreduction under continuous illumination in the absence of O_2_ (A) Evolution upon illumination of absorption at 610 nm as a function of MV^2+^ concentration. [LAC3] = [Ru] = 30 μM, [EDTA] = 10·mM, [MV^2+^] = 0–300·μM as indicated in Britton Robinson buffer pH 4.0 (25°C) in the absence of O_2_. Solid lines show the best fit of a global simulation of the absorption kinetics with the reaction model described in the supplemental information, transparent methods Section). Example of the evolution of concentrations of the different intermediates during photoreduction of laccase as deduced from the global fit procedure for a concentration of MV^2+^ of 50 μM (corresponding to the blue curve in panel A, reproduced as dotted black line). All red intermediates contribute to the absorption at 610 nm, whereas the blue intermediates do not. Note that the last reduction step corresponds to a transition T1_red_TNC^2^ → T1_red_TNC^3^ (compare dashed and solid blue lines between 230 and 420 s) meaning that T1 stays almost fully reduced. This indicates that the third reduction of the TNC from T1_red_ is endergonic (see text).

A solution of sensitizer and LAC3 containing 10 mM EDTA and different concentrations of the MV^2+^ electron relay was irradiated with a white LED (filtered, 450<*λ* < 700 nm) in the absence of O_2_, and the evolution of absorption at 610 nm was followed as a function of illumination time (Figures 3A and S7). Before illumination, the absorption at 610 nm is exclusively attributable to the oxidized (Cu^II^) T1 site. Upon illumination this absorption decreases with a concave shape to reach zero, indicating complete reduction of the Cu^II^ T1 site. With increasing concentration of the electron mediator, MV^2+^, the time interval needed for T1 reduction is strongly decreased (Figure 3A). The plot of the reduction rate of T1 (defined as the reciprocal of the time needed for complete reduction of T1) as a function of the concentration of MV^2+^ reveals a linear dependence (Figure S8) with an acceleration by a factor of >10 for a MV^2+^ concentration of 300 μM compared with the system without MV^2+^.The linear dependence can be traced back to the linear dependence on MV^2+^ concentration of the quantum yield of MV^2+^ reduction prevailing at these low concentrations (Figure S8). Rescaling of the absorption transients to match the time needed for disappearance of T1 absorption reveals very similar shapes of the kinetics in the absence and presence of MV^2+^ (for all concentrations studied, Figure S9), strongly suggesting that there is no fundamental difference in the mechanism of reduction of the T1 site whether reduction occurs directly from the excited state of the chromophore or via MV^⋅+^. After the absorption at 610 nm has decayed close to zero, indicating accumulation of reduced T1, a re-increase of absorption at this wavelength is observed (Figure 3A) that can be clearly assigned to the accumulation of reduced methyl viologen radical MV^⋅+^ because its typical absorption band at 395 nm grows concomitantly (Figure S7). Interestingly, especially at low concentrations of MV^2+^ we noted a lag time between complete T1 reduction and the start of accumulation of MV^⋅+^ (Figures 3A and S7 right). As observed for the reduction of T1, this time is shorter for higher concentrations of MV^2+^. It seems reasonable to attribute this delay to the time needed for the total reduction of laccase (four reducing equivalents). The fact that no MV^⋅+^ radical is detectable before complete laccase reduction implies that the rate of generation of the MV^⋅+^ radical species by the excited sensitizer is slower than its consumption by the laccase enzyme. To put numbers to this, at the highest MV^2+^ concentration a rate of formation of MV^⋅+^ of 2 μM/s is estimated. This means that with the employed concentration of LAC3 (30 μM) the maximum rate of photoreduction of LAC3 is < 0.067 s^−1^.

To investigate the observed kinetics, we performed a global simulation of the absorption changes recorded with different concentrations of MV^2+^ (Figure 3). The presence of four different reduction states of the laccase that can interact with two potential electron donors, [Ru(bpy)_3_]^2+^^∗^ or MV^⋅+^, makes the system rather inimical to analyze and requires some simplifying assumptions. First, it seems sound to assume that electron input into the LAC3 from either [Ru(bpy)_3_]^2+^^∗^ or MV^⋅+^ occurs via the T1 site (as supported by the laser flash data, at least for the first reduction). Furthermore, we neglect the details of reactions occurring within association complexes between [Ru(bpy)_3_]^2+^ and LAC3 and modeled laccase reduction from the sensitizer's excited state by a low-yield process. With these simplifications and because many rate constants are reasonably well known from kinetic flash photolysis measurements and are independent of MV^2+^ concentration, a reasonable fit to the experimental data could be obtained, which reproduces the major features of the photoreduction assay with a common set of parameters (solid lines in Figure 3A). With this model in hand, we can now investigate the critical parameters with the aim to extract information on the mechanism governing the photoreduction of laccase and to determinate the rate constants for the reactions describing internal electron transfer (IET) from T1 to the TNC center. In the following we will present only the major results; details of the analysis are given in the transparent methods section in the supplemental information.

**Scheme 3:**
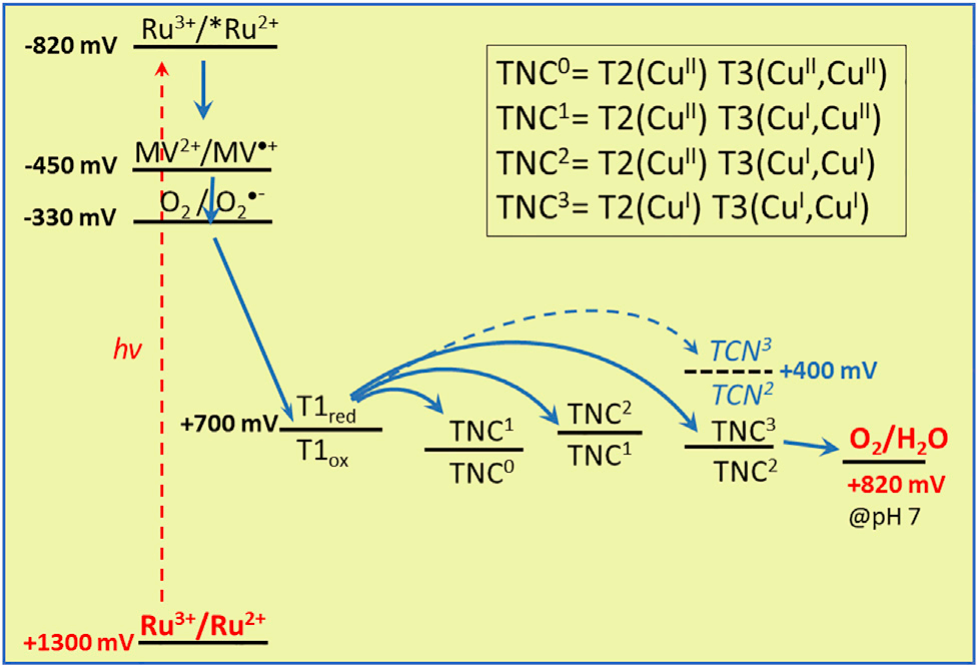
Energetic landscape of chromophore/electron relay/LAC3 system Light absorption produces the strong oxidant [Ru(bpy)_3_]^3+^ stabilized by irreversible transfer of the electron via MV^⋅+^, O_2_^⋅–^, and the laccase enzyme to O_2_. The dashed level indicates the lower potential of the TNC under anaerobic conditions.

We use the nomenclature of Sekretaryova et al. (Sekretaryova et al., 2019) to describe the redox states of laccase from fully oxidized (T1_ox_TNC^0^) to the fully reduced form (T1_red_TNC^3^; with TNC^x^, x = number of electrons on the TNC). The initial absorption decrease at 610 nm describes the loss of absorption from oxidized T1 and corresponds to the consumption of three electrons, leading to reduced T1 and doubly reduced TNC (T1_red_TNC^2^, dashed blue trace in Figure 3B). The first two IET steps from T1 to TNC, leading to the formation of T1_red_TNC^2^, are fast compared with the photoproduction of MV^⋅+^, which is the rate limiting step. Accordingly, the analysis was found to be not sensitive to the rates of forward electron transfer from T1 to the TNC, provided they are faster than about 0.1 s^−1^. We assumed for the fits rate constants of 1 s^−1^ as found in a pulse radiolysis study (Farver et al., 2011). The lag time following reduction of T1 corresponds to the uptake by the laccase of the fourth electron leading to full reduction of the enzyme (T1_red_TNC^3^, solid blue line in Figure 3B). As can be seen in the profiles of the intermediates, this last reduction step corresponds to the transition T1_red_TNC^2^ → T1_red_TNC^3^ (compare dashed and solid blue lines between 230 and 420 s) meaning that T1 stays almost fully reduced during the process although T1 is the entrance side for the electron. In fact, the kinetic parameters determined from the global fit clearly show that the third reduction of the TNC from T1_red_ is significantly endergonic (*k*_+_/*k*_-_ ≈ 1/4500; Δ*G* ≈ +200 meV), in contrast to the first two IET steps that are close to equilibrium (Scheme 3). Because during catalytic turnover all internal electron transfer steps leading to full reduction of the oxidized native intermediate (NI) are fast, even in high-potential laccases as LAC3 used in our study^,^(Sekretaryova et al., 2019; Heppner et al., 2014; Solomon et al., 1996), we attribute this sluggish third-electron reduction of the TNC to the fact that under the anaerobic conditions of this experiment the oxidized enzyme is not in its native intermediate form but in the resting oxidized (RO) state well described in the literature (Tollin et al., 1993). The characteristics of internal electron transfer deduced from our experiment are in agreement with results from pulse radiolysis and direct electrochemical measurements reporting a potential of the T2 Cu site in the RO state of around 400 mV versus NHE, i.e. about 300 mV lower than the potentials of T1 and T3 (Farver et al., 2011; Shleev et al., 2005). Although there is “insufficient driving force” for ET from reduced T1 to T2, our results show that electron transfer occurs because the overall energetics is still favorable due to the low potential of the MV^⋅+^ “substrate.” The possibility of uphill intramolecular electron transfer under certain conditions had also been proposed based on electrochemical and quantum chemical studies (Shleev et al., 2012). Furthermore, our conclusions fit with observations of Lazarides et al. on laccase photoreduction by a porphyrin chromophore in presence of EDTA where it was found that the ESR signal of the T1 Cu(II) site disappeared much faster (30 s) than the signal of the T2 Cu(II) species (30 min) (Lazarides et al., 2013). It appears that monitoring the accumulation of the reduced MV^⋅+^ radical is an indirect but valuable tool to obtain information about IET events within the laccase enzyme, complementing measurements of absorption changes related to the oxidation state of T1, which are the only observables in the absence of MV^2+^. Interestingly, the consistent analysis performed on this set of experiments, i.e., laccase photoreduction in the absence and the presence of MV^2+^, reveals that in the absence of MV^2+^, where laccase reduction occurs via the excited state of the chromophore, the enzyme is only reduced by three electrons (Figure S10). The reason for this is the unfavorable pre-equilibrium resulting from the endergonic nature of the third reduction of the TNC, which makes the IET T1_red_TNC^2^ → T1_ox_TNC^3^ too slow to be compatible with the short lifetime of the [Ru(bpy)_3_]^2+^^∗^ excited state. In contrast, the MV^⋅+^ species is long-lived and can progressively reduce the state T1_ox_TNC^3^ present at low concentration in the above-mentioned equilibrium with T1_red_TNC^2^ to form T1_red_TNC^3^. Of note, in the presence of dioxygen where all internal electron transfer steps are nearly isoenergetic, the complete, four-electron reduction of laccase via [Ru(bpy)_3_]^2+^^∗^ is possible.

Finally, to obtain a satisfactory global fit *of* the absorption transients describing accumulation of MV^⋅+^ after full, four-electron reduction of the enzyme (Figure 3A), we had to include in the simulation a feature that accounts for a lack of the quantity of MV^⋅+^ accumulating (reaction A15 in the supplemental information, transparent methods Section). The missing quantity, most clearly visible in the traces for low concentration of MV^2+^ (see Figure S9), corresponds roughly to the quantity of enzyme present (30 μM). We therefore tentatively attribute this feature to the formation of adducts of MV^2+^, with the enzyme being most efficient when LAC3 becomes fully reduced. The “bound” MV^2+^ might still be reducible by [Ru(bpy)_3_]^2+^∗ but the dissociation rate for MV^⋅+^ or Ru^3+^ from the Ru^3+^:MV^⋅+^:LAC3 complex might be too low to avoid charge recombination between MV^⋅+^ and Ru^3+^.

### Dioxygen consumption

**Figure 4:**
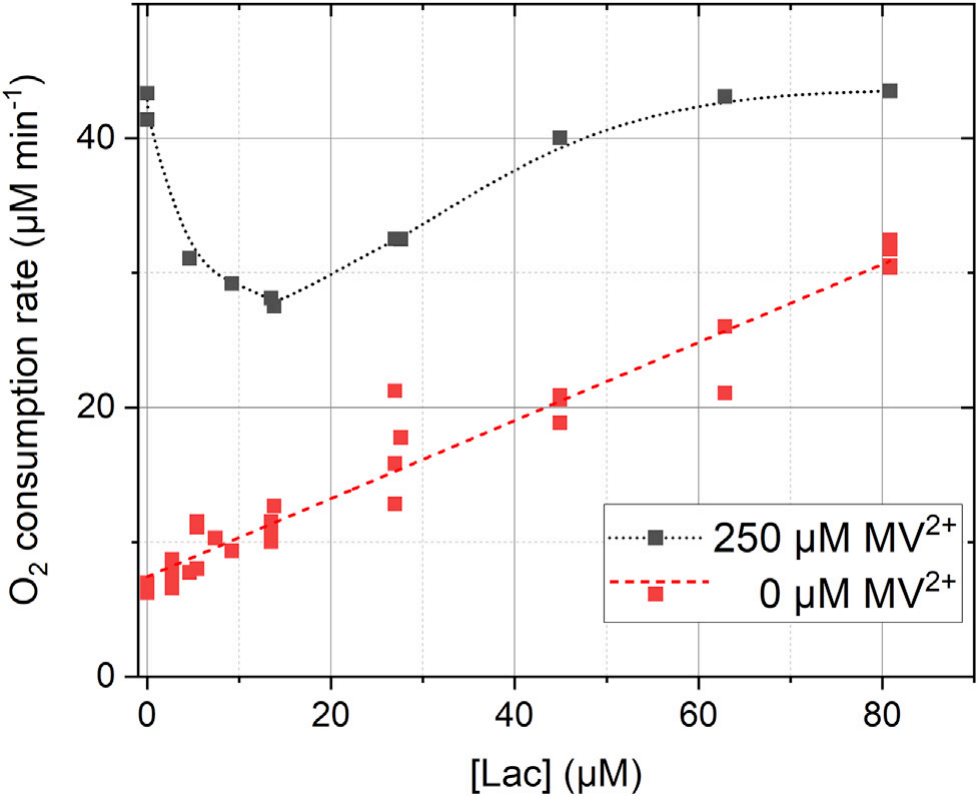
Rate of light-induced dioxygen consumption as a function of laccase concentration [Ru] = 30·μM, [EDTA] = 10·mM, [MV^2+^] = 0 or 250·μM, [LAC3] = 0–81·μM in Britton Robinson buffer pH 4.0 (25°C). Initial dissolved oxygen concentration was 250 μM.

**Figure 5:**
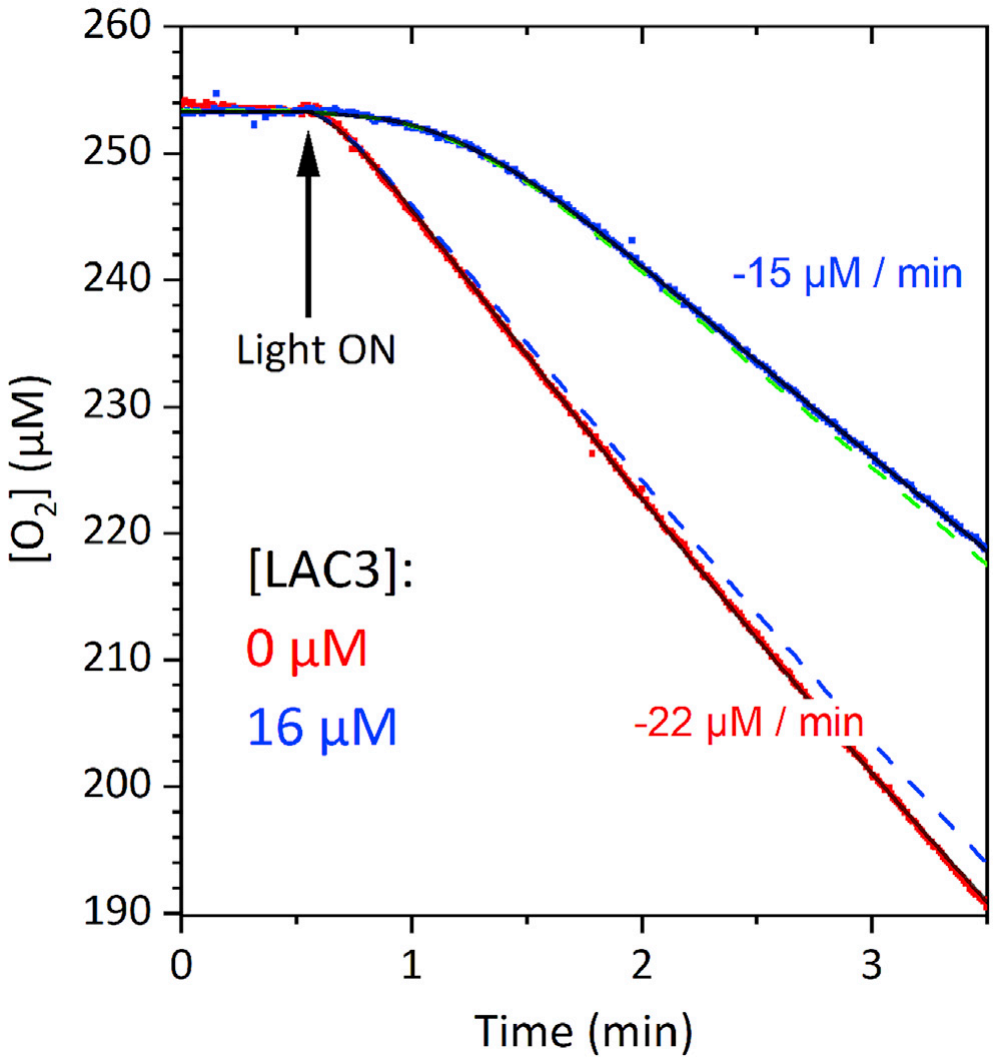
Light-induced oxygen consumption profile of a solution of 30 μM [Ru(bpy)_3_]^2+^, 1 mM EDTA, and 1 mM MV^2+^ in B&R buffer pH 6, in the absence (red trace) and presence (blue trace) of 16 μM LAC3 Best fits (black solid lines) superimposed with the experimental data. The fit was performed according to a reaction scheme considering adduct formation between O_2_^⋅−^ and LAC3 followed by slow internal ET (for fit parameters see supplemental information, transparent methods Section). Dashed blue line: simulation of O_2_ consumption in presence of LAC3 but without considering reduction of LAC3 by O_2_^⋅−^ (*k*_D9-D12_ = 0) keeping all other parameters constant. Dashed green line: simulation of O_2_ consumption in the presence of LAC3 but without considering reduction of LAC3 by MV^⋅+^ (*k*_D5-D8_ = 0) keeping all other parameters constant. Comparing the dashed lines with the solid blue line indicates that O_2_^⋅−^, and not MV^⋅+^, is the predominant reductant for the laccase under these conditions. See Figures S12 and S13 in the supplemental information for further discussion.

With the finding that the presence of an electron mediator helps transferring electrons faster and more efficiently to the LAC3, we then performed a set of dioxygen consumption experiments to characterize the catalytic activity of the laccase when reduced by a sensitizer/electron mediator/electron donor photoredox system. Under these aerobic conditions, O_2_ competes with the laccase for the electron from the MV^⋅+^ species as it is well known that in aqueous solution MV^⋅+^ reacts rapidly with O_2_ to form O_2_^⋅–^ (or O_2_H^⋅^ at our operating pH) (Farrington et al., 1973). This competition between laccase and O_2_ is clearly evident from transient absorption measurements revealing a markedly reduced efficiency of flash-induced T1 reduction with increasing amounts of O_2_ (Figure S11). Surprisingly though, when light-induced dioxygen consumption was measured with a Clark electrode, the effect of laccase was much stronger than expected from this simple competition scheme. Figure 4 shows the rate of O_2_ consumption as a function of LAC3 concentration in the absence and presence (250 μM) of MV^2+^. In the absence of MV^2+^, the rate increases linearly with the concentration of LAC3, a behavior easily explained by an increased reduction of laccase from the excited state of the chromophore due to increased formation of association complexes (see above). When MV^2+^ is present, but no laccase, a fast dioxygen consumption rate is observed, which corresponds to the two-electron reduction of O_2_ to H_2_O_2_ via dismutation of O_2_^⋅–^ (eqs. B and D, Figure 5 red trace). Addition of LAC3 at a concentration much lower than O_2_ (10–20 μM of LAC3 compared with 250 μM O_2_) leads to a very significant reduction of the dioxygen consumption rate (Figure 4, black curve; Figure 5 blue trace).

(B) MV^⋅+^ + O_2_ → MV^2+^ + O_2_^⋅–^

(C) 4 MV^⋅+^ + 4 Cu^II^ → 4 MV^2+^ + 4 Cu^I^

(D) 2 O_2_^⋅–^ + 2 H^+^ → O_2_ + H_2_O_2_

(E) 4 O_2_^⋅–^ + 4 Cu^II^ → 4 O_2_ + 4 Cu^I^

(F) 4 Cu^I^ + O_2_ + 4H^+^ → 4 Cu^II^ + 2 H_2_O

2 MV^⋅+^ + O_2_ + 2 H^+^ → 2 MV^2+^ + H_2_O_2_ (B)+(D)

4 MV^⋅+^ + O_2_ + 4H^+^ → 4 MV^2+^ + 2 H_2_O (C)+(F) or 4x(B)+(E)+(F)

**Scheme 2:**
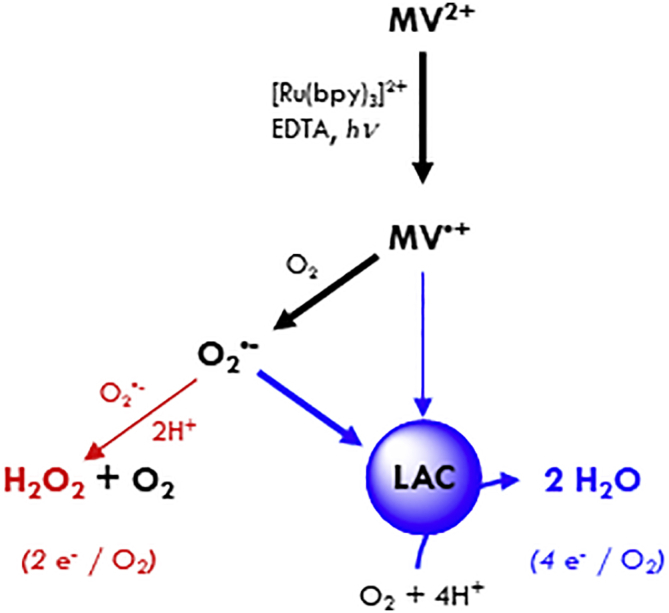
Dioxygen reduction routes in the absence (2e^−^/O_2_, red) or presence (4e^−^/O_2_, blue) of laccase Thickness of arrows indicates predominance of pathways. The data on the effect of LAC3 on O_2_ consumption rate in Figure 4 (pH 4) and Figure 5 (pH 6) both demonstrate the participation of the enzyme, suggesting that even at pH 4 O_2_^⋅–^ is sufficiently long lived to reduce laccase.

When laccase accepts electrons, a four-electron/four-proton total reduction of O_2_ is performed by the enzyme (eqs. C and F) in addition to the two-electron/two-proton partial direct reduction of O_2_ to H_2_O_2_ (eqs. B and D). A substantial addition of an enzyme consuming half as much O_2_ for the same concentration of photogenerated MV^⋅+^ could thus show up in the dioxygen consumption curve as a decrease in the dioxygen consumption rate compared with MV^2+^ alone. Therefore, the pattern in Figure 4 can be explained by a competitive scheme where O_2_ is being progressively substituted by LAC3 as electron recipient from MV^⋅+^ because the two paths distinctively produce end products, either H_2_O_2_ or H_2_O (Scheme 2). When enough laccase is present to compete with O_2_ for electrons from MV^⋅+^ the rate increases and reaches a saturation value given by the light intensity and efficiency of the sacrificial electron donor. However, the effect of laccase on the rate of O_2_ consumption at laccase concentrations much lower than the concentration of dissolved O_2_ (250 μM) is surprisingly pronounced. This suggests that laccase reduction is more efficient than expected from a simple competition between laccase and O_2_ for electrons from MV^⋅+^ as observed in Figure S11. We therefore propose that laccase can be reduced not only by MV^⋅+^ but also and quite significantly by the O_2_^⋅–^ formed via electron transfer from MV^⋅+^ to O_2_ (eq. 4xB, E). Analysis of the O_2_ consumption kinetics in the absence and presence of laccase (Figures 5 and S12) supports the interpretation that it is indeed O_2_^⋅–^ that is the dominant reductant for the laccase under these conditions. Laccase reduction by O_2_^⋅–^ had also been reported based on pulse-radiolysis studies (Guissani et al., 1982; (Pecht et al., 1977)). The two processes (eqs. (C)+(F) or 4x(B)+(E)+(F)) should then significantly prevent an accumulation of H_2_O_2_. As a matter of fact, H_2_O_2_ concentration dropped dramatically as laccase was introduced in dioxygen consumption experiments (Figure S14). The reaction sequence for photoreduction of laccase under aerobic conditions is summarized in Scheme 2.

However, if O_2_^⋅–^ can replace MV^⋅+^ for reduction of LAC3 as suggested by the O_2_ consumption experiments, then the question arises why is the bleaching of T1 absorption, as detected in the laser flash experiment (Figure S11), strongly reduced in the presence of O_2_? To answer this question, we performed a global kinetic analysis of the oxygen consumption profile in the absence and presence of laccase (Figure 5). It is interesting to note that a first analysis of the O_2_ consumption kinetics (see Figure S12 and transparent methods Section in the supplemental information) supports a slow reaction of superoxide with LAC3 with a rate not exceeding 5 × 10^6^ M^−1^s^−1^ (yielding a time constant of >1 ms for a concentration of O_2_ of 250 μM), about two orders of magnitude slower than the rate of laccase reduction by MV^+⋅^ (see legend of Figure 2 and Scheme S2 in the supplemental information). If this applies also for the flash-induced process, T1 reduction would be too slow to be detected on the timescale of the transient absorption measurement (1 ms) but could be still fast enough to be operational under the continuous light conditions of the dioxygen consumption assay. Such a slow reduction of T1 by O_2_^⋅–^ has been described in pulse radiolysis studies where laccase was shown to be an efficient radical scavenger (Guissani et al., 1982; Goldberg and Pecht, 1978). In these studies, O_2_^⋅–^, as other reducing radicals, was found to interact with the laccase not through a direct reduction of the T1 center but instead by fast formation of a transient adduct with the protein followed by slow intra- or intermolecular electron transfer from the adduct site to the T1. A refined kinetic simulation shows that such a scenario is fully compatible with the kinetics of O_2_ consumption shown in Figure 5, provided that the adduct formation is reversible (see transparent methods Section in the supplemental information). This scenario can account for the two orders of magnitude difference in the apparent bimolecular rate constants for laccase reduction via MV^⋅+^ or O_2_^⋅–^ in a more convincing way because such a difference in bimolecular interaction rates would be difficult to rationalize. Unfortunately, neither the exact locus of the O_2_^⋅–^ adduct nor a definite mechanism for the intramolecular electron transfer have been determined in these pulse radiolysis investigations. Although not favored by the authors of these earlier studies, it is tempting to hypothesize on the possibility of O_2_^⋅–^ interacting with the enzyme at the level of the TNC considering the fact that this is the natural binding site for O_2_ in laccases. The data presented here do not allow to draw conclusions on this topic, but they might give indications for experiments dedicated to address these questions. Importantly though, our results clearly evidence the specificity of the enzyme acting as a sink for photogenerated electrons that it can efficiently recover either from an electron mediator or from a superoxide radical and use them for the reduction of O_2_ to H_2_O. To visualize the action of laccase Figure S13 shows the evolution of the concentration of key species during the photocatalytic cycle.

### Conclusion

Using synthetic chromophores to initiate light-driven electron transfer processes for photo-biocatalysis can provide unique solutions to perform green chemical transformations. However, an innate limitation in elaborating such systems resides in the presence of unproductive energy transfer processes that can outcompete ET processes (Winkler, 2013). In this study, we clearly show that detrimental energy transfer can be short circuited by the addition of a reversible electron acceptor, which transfers electrons from [Ru(bpy)_3_]^2+∗^ to a laccase with high quantum yields. Even when the electron relay is reducing enough to interact with O_2_ to form superoxide radicals, the laccase enzyme can efficiently accept electrons from the latter species to catalyze O_2_ reduction and thereby short circuit the formation of H_2_O_2_. In this way O_2_ functions as both, an electron shuttle to and oxidizing substrate for the laccase, leading to an efficient photocatalytic cycle. With the presented thorough description of the charge transfer and dioxygen reduction steps involving this MCO, we show that the dioxygen from air, usually carefully eliminated from reaction media in synthetic systems mimicking photosynthesis, can become a key element in photo-catalysis as a safe, renewable, and inexpensive alternative to sacrificial electron acceptors. In parallel to the consumption of O_2_ in such systems, a powerful oxidant, [Ru(bpy)_3_]^3+^ (1300 mV versus NHE), is photo produced, ready to drive the oxidation of more tenacious organic substrates (Scheme 3). Among future challenges ahead of us lie the use of the oxidative power of the oxidized chromophore for further chemical transformations in lieu of the sacrificial electron donor used in this study. Work along these lines is underway in our labs. In a broader context, this study also highlights the potential of light-controlled electron delivery to enzymes as an interesting alternative to stopped-flow techniques to reveal functional details of internal charge transfer and charge accumulation at the catalytic sites. The interested reader is referred to Figure S15 in the transparent methods section of the supplemental information for some illustration.

### Limitations of the study

This work provides insight on coupling of laccase with a photoredox system to create highly oxidizing species using dioxygen as terminal electron acceptor. As the next step we are working on the application of this system for oxidative catalysis by replacing the sacrificial electron donor EDTA used in this study by a catalytic system for oxygen atom transfer reactions. Further work is required to analyze the effect of pH on both the efficiency of laccase reduction and the oxidation catalytic cycle.

### Resource availability

#### Lead contact

Further information and requests for resources should be directed to and will be fulfilled by the lead contact, Winfried Leibl (winfried.leibl@cea.fr).

#### Materials availability

This study did not generate new unique reagents.

#### Data and code availability

The published article includes all datasets/code generated or analyzed during this study.

## Methods

All methods can be found in the accompanying transparent methods supplemental file.

## Supplemental information

Document S1. Transparent methods, figures S1–S15, and scheme S1 and S2

## Footnotes

Supplemental information can be found online at https://doi.org/10.1016/j.isci.2021.102378.
