# Supplementary material for: Tracking light-induced electron transfer toward O2 in a hybrid photoredox-laccase system
Source: iScience. 2021 Mar 31;24(4):102378. doi: 10.1016/j.isci.2021.102378 (PMC8080520; doi:10.1016/j.isci.2021.102378)
Supplement: Document S1. Transparent methods, figures S1–S15, and scheme S1 and S2 [file mmc1.pdf]

iScience, Volume *24*

Supplemental information

Tracking light-induced electron transfer toward O_2 in a hybrid photoredox-laccase system

Rajaa Farran, Yasmina Mekmouche, Nhat Tam Vo, Christian Herrero, Annamaria Quaranta, Marie Sircoglou, Frédéric Banse, Pierre Rousselot-Pailley, A. Jalila Simaan, Ally Aukauloo, Thierry Tron, and Winfried Leibl

Supplemental Figures

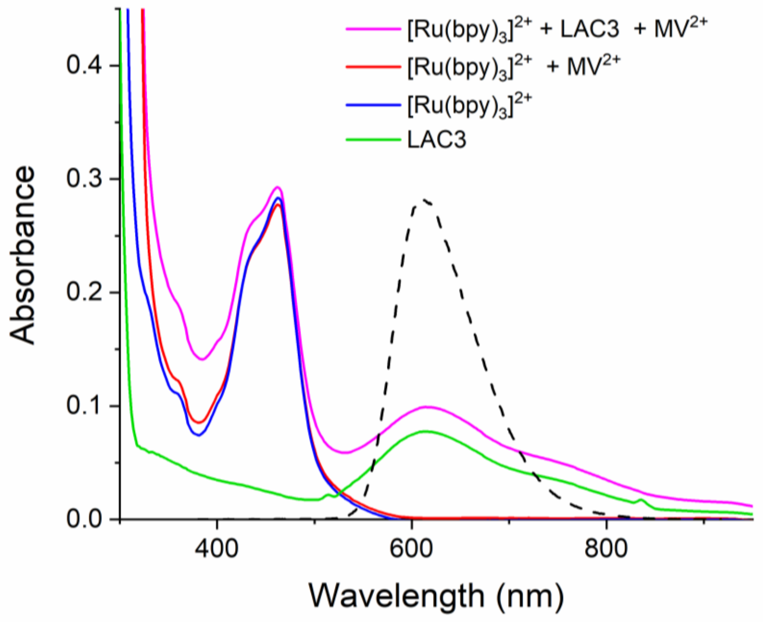

Figure S1. Absorption spectra of samples recorded in B&R buffer, pH = 6. Concentrations: $[Ru(bpy)_3]^{2+}$ (17 µM), LAC3 (17 µM), MV^{2+} (10 mM). The dashed line shows the emission spectrum of $[Ru(bpy)_3]^{2+}$*. Related to Fig. 1, Fig. 2, and Fig. 3.

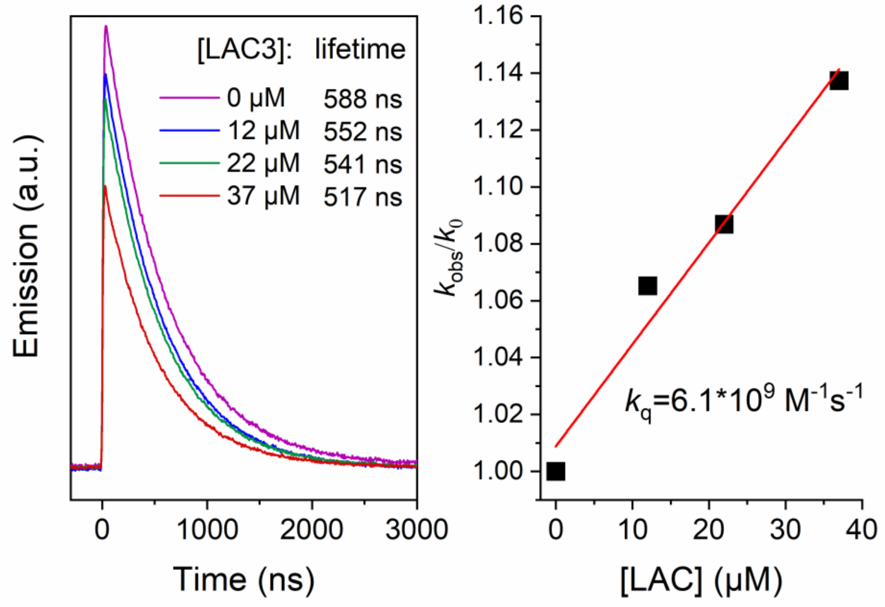

Figure S2. Quenching of $[Ru(bpy)_3]^{2+*}$ emission by laccase. Left: Emission decay at 610 nm for a solution of $[Ru(bpy)_3]^{2+}$ (15 µM) and different concentrations of LAC3 in B&R buffer, pH = 6 after excitation at 455 nm. Right: Stern-Volmer plot derived from monoexponential fits to the data. The decrease in amplitude of the emission in presence of laccase is mainly attributable to an inner filter effect caused by the absorption of the oxidized laccase overlapping the spectral region of $[Ru(bpy)_3]^{2+}$ emission (see Fig. S1). For the highest LAC3 concentration part of decrease in initial emission amplitude is due to a dilution effect. Related to Fig. 1 and Fig. S1.

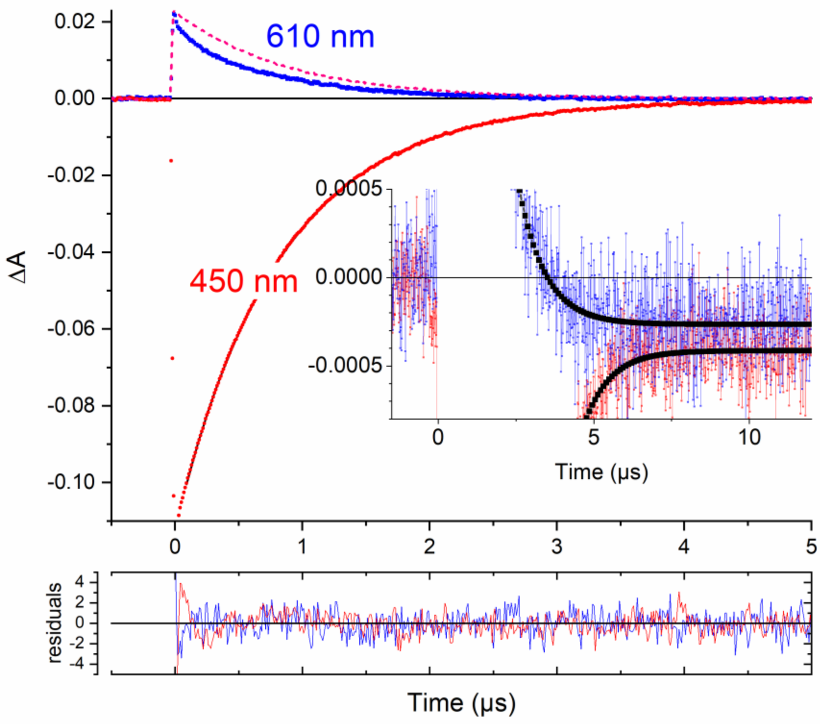

Figure S3. Transient absorption kinetics of a solution of $[Ru(bpz)_3]^{2+}$ (15 µM) and LAC3 (36 µM) in B&R buffer, pH = 5.7 after laser flash excitation at 455 nm. Inset: zoom of the data at long times. Bottom: residuals for best fit according to the reaction scheme described in Scheme S1 below. The dashed pink line is the scaled kinetics of emission at 610 nm indicating that the 610 nm transient contains contribution from other species than the excited state. Related to Fig. 1 and Scheme S1.

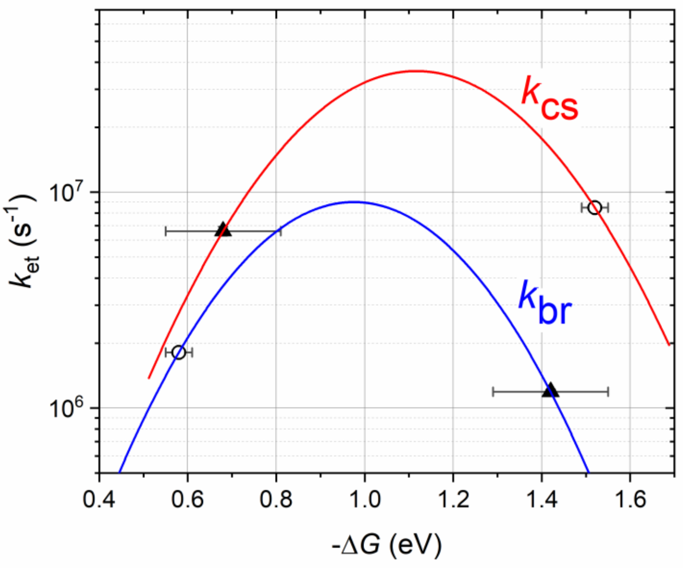

Figure S4. Plot of the first order rate constants of electron transfer in sensitizer-LAC3 complexes (eq. (A). Charge separation from the excited state (red, k_{cs}) and charge recombination to the oxidized sensitizer (blue, k_{br}). Sensitizers $[Ru(bpy)_3]^{2+}$ (open circles) or $[Ru(bpz)_3]^{2+}$ (filled triangles). Solid lines show best fits to the Marcus equation (see section on calculations below) yielding λ_{cs} = 1.12 eV, λ_{cr} = 0.97 eV, r_{cs} = 12.5 Å, and r_{cr} = 13.5 Å as values for the reorganization energy and donor-acceptor distances for charge separation and recombination. Related to Fig. 1, Fig. S3, Scheme 1, and Scheme S1.

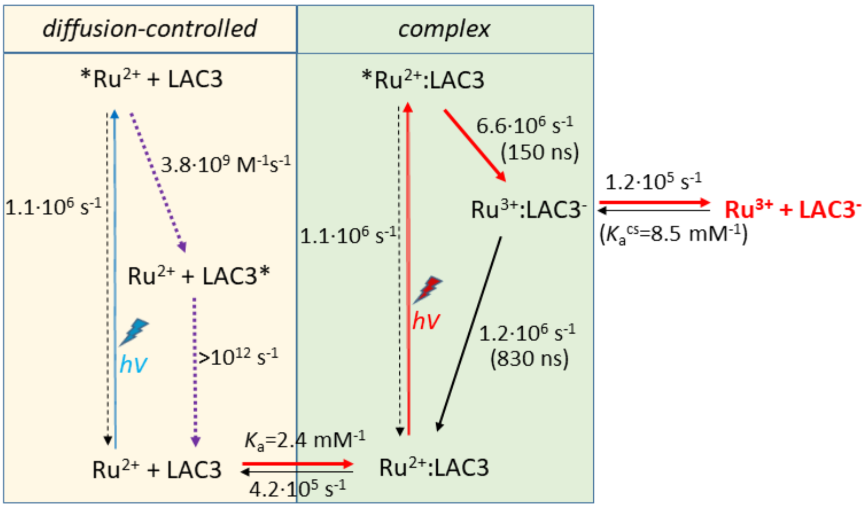

Scheme S1. Interactions between the $[Ru(bpz)_3]^{2+}$ photosensitizer and the LAC3 enzyme identified by laser flash photolysis experiments. Reactions leading to formation of reduced laccase ($LAC3^-$) and oxidized photosensitizer (Ru^{3+}) are indicated by red arrows. In the bimolecular pathway (left) Förster resonance energy transfer quenching of the $[Ru(bpz)_3]^{2+*}$ excited state largely outcompetes with electron transfer to the T1 Cu^{II} center in LAC3. In the association complex (right) charge recombination is about 10 times faster than dissociation leading to a low yield for formation of the target state Ru^{3+} $LAC3^-$ (red). Parameter values were deduced from analysis of the data of Fig. S3 (pH 5.7). Related to Fig. 1, Fig. S3, and Scheme 1.

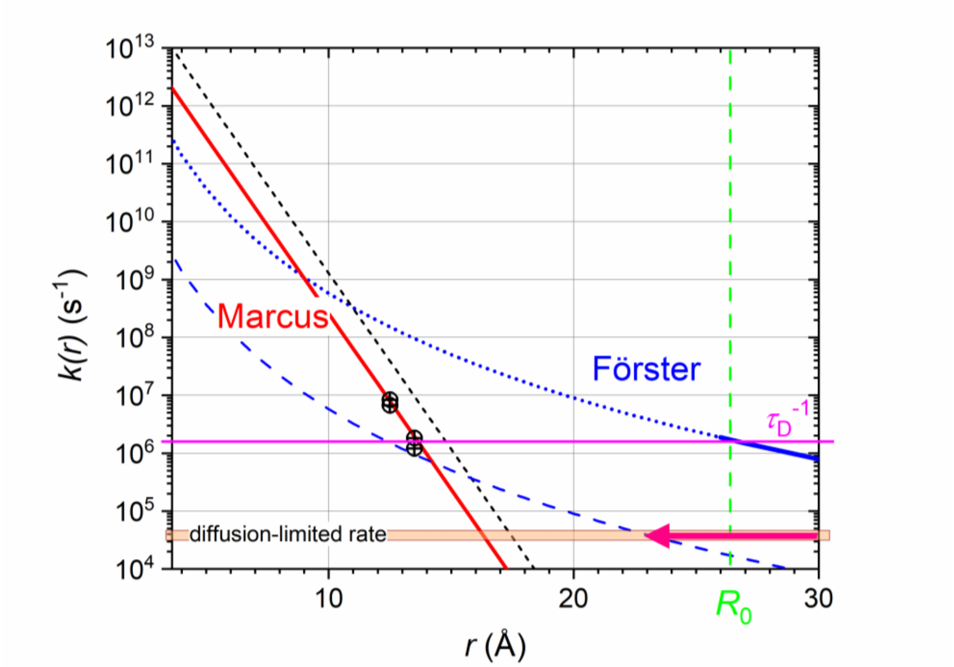

Figure S5. Rates for Förster energy transfer (blue, Eq. 1) and electron transfer (red, Eq. 4) as a function of donor-acceptor distance *r*. For the electron transfer rate, values for charge separation ($-\Delta G$ = 1.52 eV and λ = 1.12 eV) from Fig. S4 were used and the experimental rates for ET between the two chromophores and LAC3 are indicated by dots. The black dashed line represents the maximal electron transfer rate ($-\Delta G = \lambda$). The dashed blue line shows energy transfer rates 100 times slower than the Förster rates to account for a hypothetical effect of selection rules related to the triplet/doublet spin multiplicity (see text). The value of the Förster radius R_0 = 26.4 Å is represented by the dashed green line. See Methods Section for equations and details.

At the employed concentrations of LAC3 the mean distance between the photosensitizers and LAC3 are larger than 30 Å. If the position of LAC3 is taken at r = 0 Å a photosensitizer approaching (from the left side of the graph) will have to cross a $k(r)$ curve providing a rate faster than the diffusion limit for an interaction to occur. This occurs at about 23 Å in the case of "slow" Förster energy transfer and at much larger distance for normal Förster energy transfer. At these large distances Marcus ET is many orders of magnitude slower and therefore cannot occur. Related to Fig. 1, Scheme 1, Fig. S3, and Scheme S1.

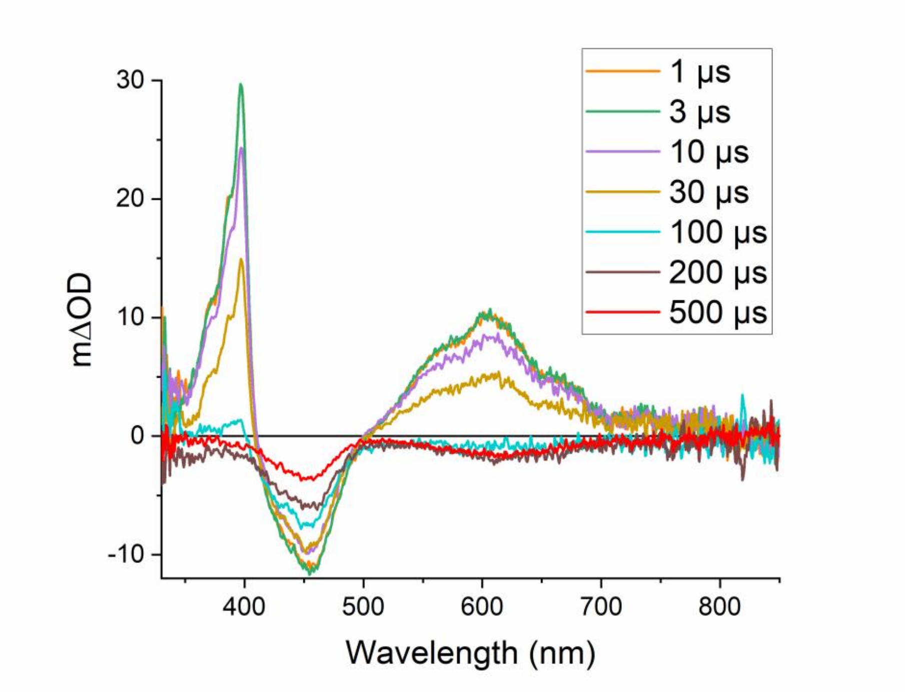

Figure S6. Transient absorption spectra of a solution of $[Ru(bpy)_3]^{2+}$ (24 µM), MV^{2+} (10 mM) and LAC3 (30 µM) in B&R buffer, pH =4 recorded at different delay times after laser flash excitation at 460 nm. Related to Fig. 2.

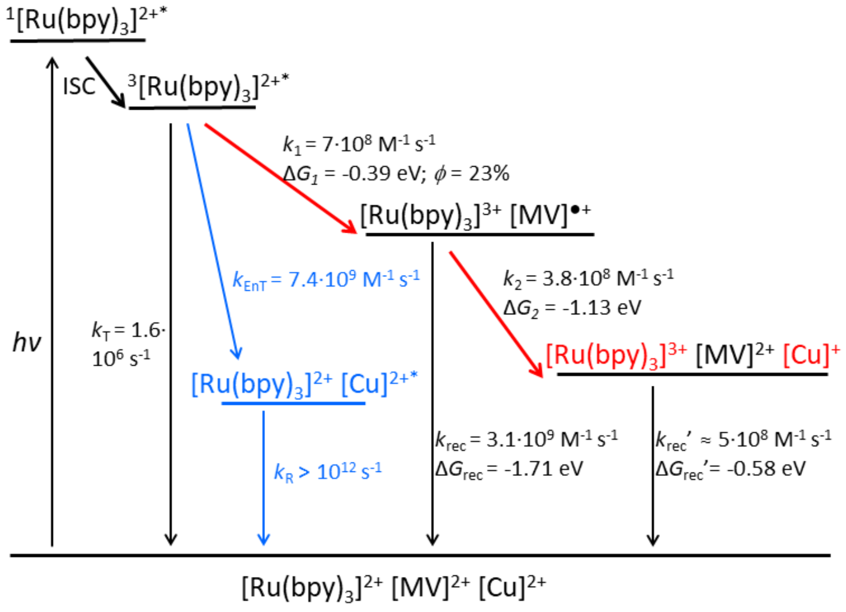

Scheme S2. Reaction scheme displaying energetic and kinetic characteristics after laser flash excitation of the trimolecular $[Ru(bpy)_3]^{2+}$ / MV^{2+} / LAC3 system. At high concentrations of MV^{2+} compared to LAC3 (10 mM vs 30 µM) the redox reaction between the $[Ru(bpy)_3]^{2+}$* excited state and MV^{2+} outcompetes energy transfer quenching to the T1 Cu^{II} center in LAC3. The yield of formation of $[Ru(bpy)_3]^{3+}$ and T1 $[Cu^{I}]$ then only depends on the competition between the forward electron transfer from $MV^{\bullet +}$ to LAC3 and the charge recombination of the $[Ru(bpy)_3]^{3+}$ $MV^{+\bullet}$ CSS, which clearly favors the LAC3 route even for low concentrations of LAC3. The total quantum efficiency, limited by the escape yield of MV^{2+} for formation of the charge-separated state, could be improved using a bichromophoric sensitizer (Wilson et al., 1998). Related to Fig. 1, Fig. 2, Fig. S2, and Fig. S3.

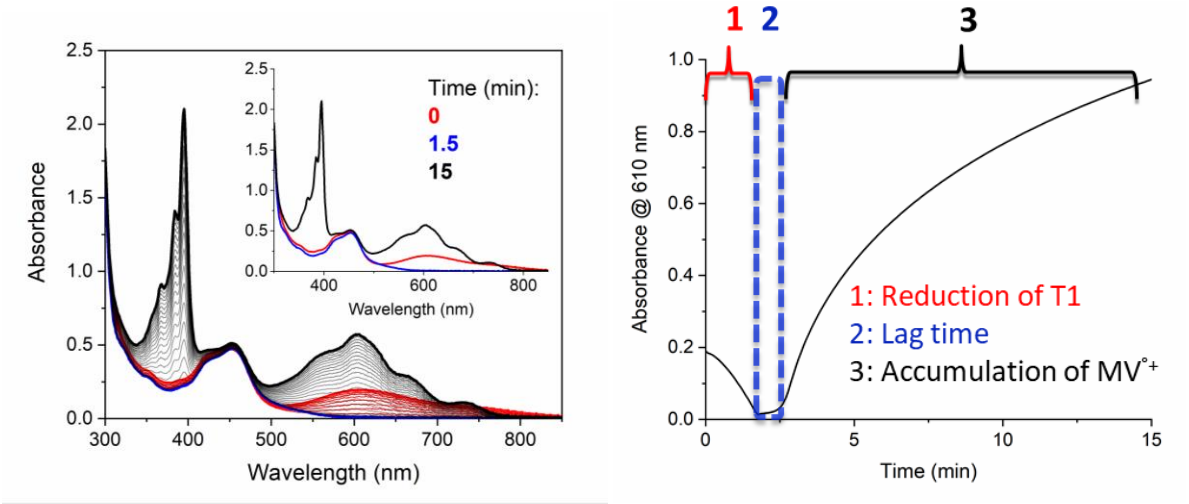

Figure S7. Evolution of absorption spectra during continuous illumination of $[Ru(bpy)_3]^{2+}$ (30 µM), LAC3 (30 µM), MV^{2+} (150 µM), and EDTA (10 mM) under anaerobic conditions. Left, Inset: absorption spectra at the indicated delay after onset of illumination. The initial absorption spectrum (red) shows contributions from $[Ru(bpy)_3]^{2+}$ (λ=450 nm) and the Cu^{II} T1 site of laccase in its oxidized form (λ=500-700 nm). After 1.6 min of illumination the 610 nm absorption band has disappeared leaving $[Ru(bpy)_3]^{2+}$ absorption (λ=450 nm, blue). After a lag phase without spectral changes the typical spectrum of $MV^{+\bullet}$ radical (395 nm, 605 nm) starts growing (black). Right: Characteristics of the time dependence of absorption changes at 610 nm with a phase of Cu^{II} T1 reduction (1) followed by a lag phase (2) and accumulation of $MV^{+\bullet}$ radical (3). Related to Figure 3.

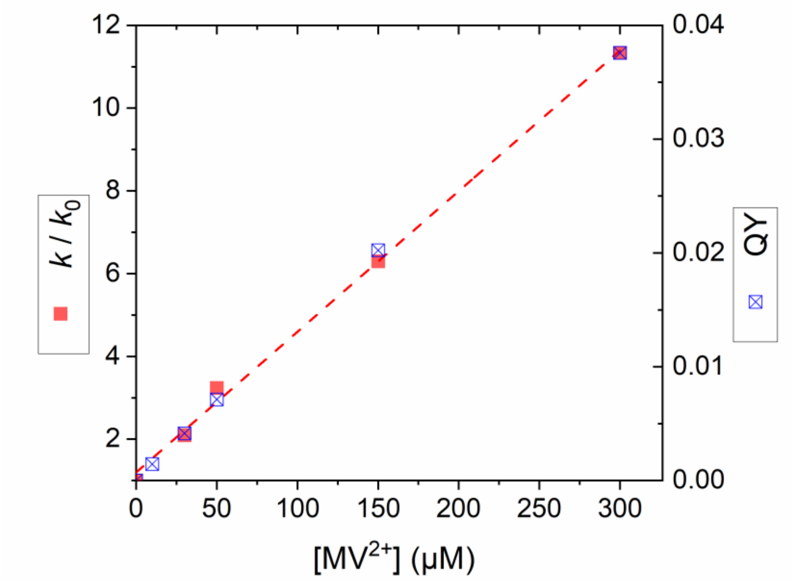

Figure S8. Effect of MV^{2+} concentration on the rate of photoreduction of T1 in a $[Ru(bpy)_3]^{2+}$/EDTA/MV^{2+} system. Rates k were taken as the reciprocal of the time of illumination needed for the total bleach of the absorption at 610 nm. k_0 is the rate in absence of MV^{2+}. Blue symbols indicate the simulated quantum yield of MV^{2+} reduction from the chromophore excited state which occurs in competition with the intrinsic decay of the excited state, considering the escape yield for $MV^{\bullet+}$ of 20%. The good correlation shows that laccase reduction is controlled by the yield of photoproduction of $MV^{\bullet+}$. Related to Figure 3A.

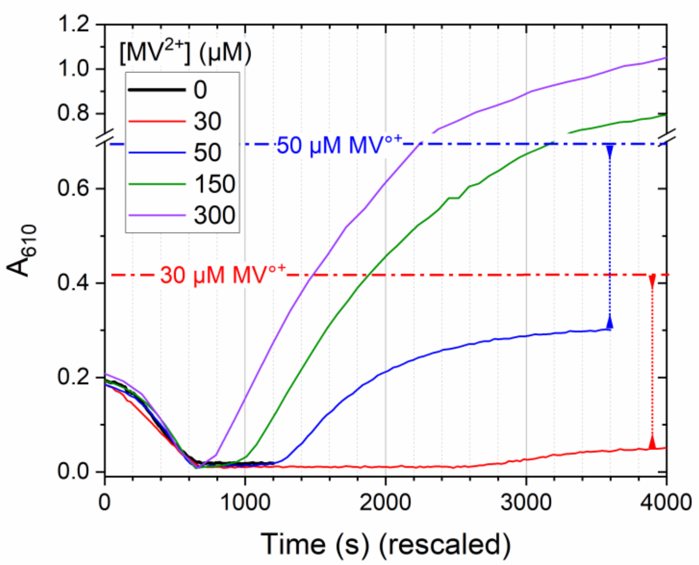

Figure S9. Laccase photoreduction under continuous illumination in absence of O_2 for increasing concentrations of MV^{2+}. Data of Fig. 3A with rescaled time axis to match the time for complete reduction of T1 for any concentration of MV^{2+} with that in the absence of MV^{2+} (for which complete reduction occurs at 620 s). The similar time courses of T1 reduction suggest the same mechanism in the absence and in the presence of MV^{2+}. The dash-dotted horizontal lines for the two lowest MV^{2+} concentrations indicate the absorption expected after complete accumulation of reduced MV^{2+} which do not match with the final absorption of the red and blue experimental traces. Related to Fig. 3.

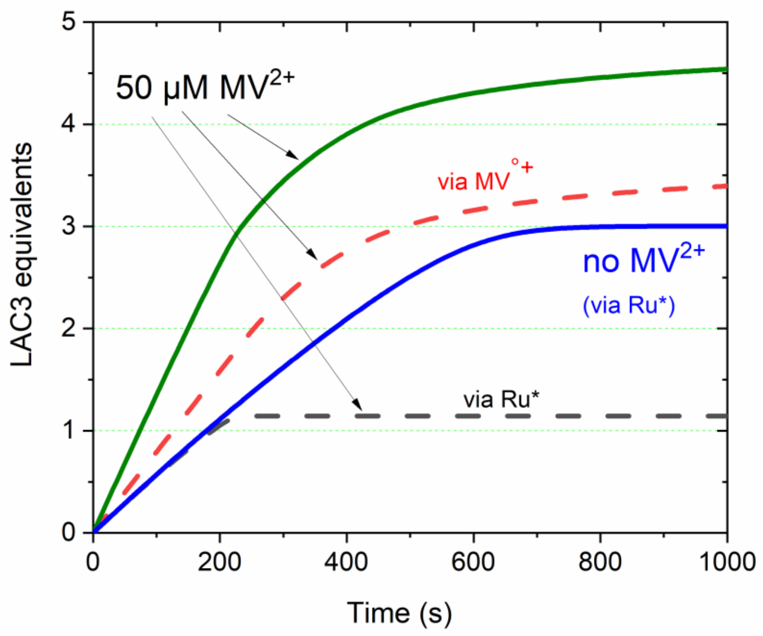

Figure S10. Plot of the time course of oxidation of the sacrificial electron donor (EDTA) in the absence of MV^{2+} (blue curve) and in the presence of 50 µM MV^{2+} (green curve). Reaction scheme and rate constants identical to those determined from the global fit of absorption transients at 610 nm (Fig. 3A). Only three electrons per LAC3 are mobilized in the absence of the electron mediator. In the presence of MV^{2+} three electrons are quickly consumed (in about 200 s), followed by a slower fourth reduction and additional consumption of electrons due to accumulation of $MV^{+\bullet}$. The dashed lines show for the case of presence of 50 µM MV^{2+} the partition of electron flux to laccase either via the excited state of the chromophore or via the electron mediator. For higher concentrations of MV^{2+} ET from Ru* becomes negligible. Related to Fig. 3.

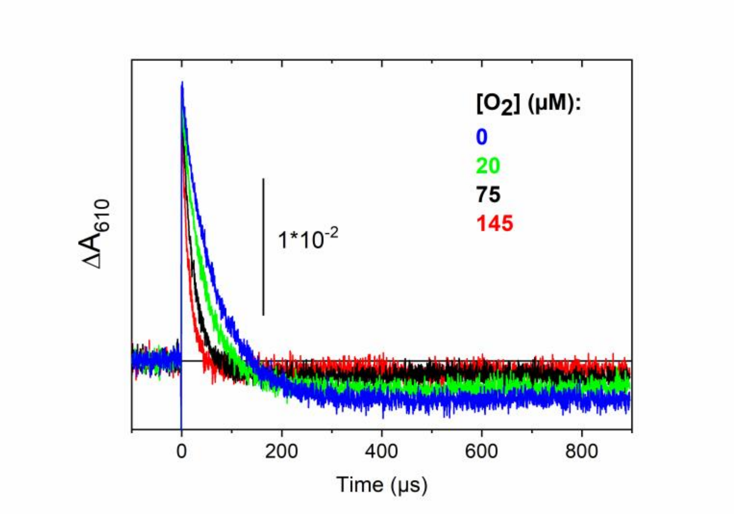

Figure S11. Transient absorption kinetics after laser flash excitation of $[Ru(bpy)_3]^{2+}$/$[BV^{2+}]$/LAC3 in B&R buffer, pH 6 recorded at 610 nm under anaerobic conditions (blue) and in presence of increasing amounts of O_2 (green, black, red). The decay indicates kinetics of $BV^{\bullet+}$ oxidation, the extent of negative absorption at longer times indicates degree of laccase reduction (bleaching of T1 absorption). The experiment was performed using benzyl viologen (BV^{2+}) instead of methyl viologen as electron relay. The less negative redox potential of the $BV^{2+}/BV^{\bullet+}$ couple compared to $MV^{2+}/MV^{\bullet+}$ leads to a decrease of the second order rate constant for electron transfer to O_2 by a factor of 2. Analyses of the viologen radical decay kinetics in aerated aqueous solution yields bimolecular rate constants for ET to O_2 of $6.6\cdot10^8$ $M^{-1}s^{-1}$ and $3.3\cdot10^8$ $M^{-1}s^{-1}$ for MV^{2+} and BV^{2+}, respectively. Related to Figure 2 and Scheme 2.

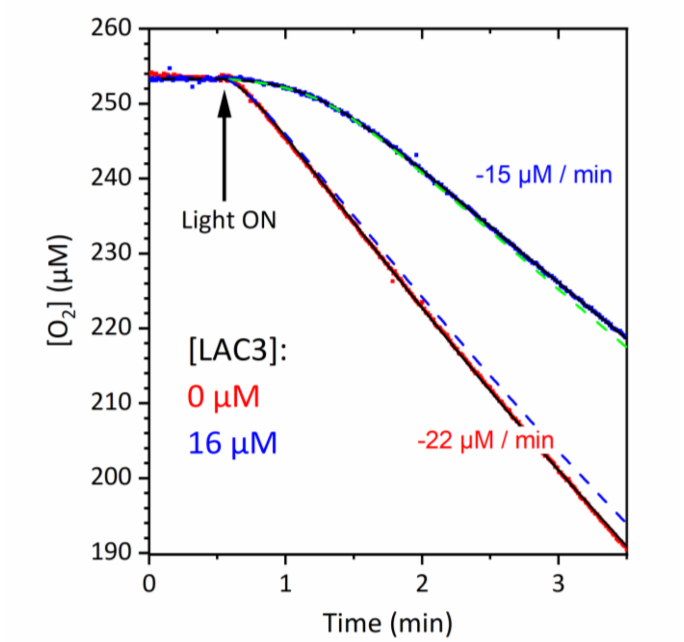

Figure S12. Light-induced oxygen consumption of a solution of 30 µM $[Ru(bpy)_3]^{2+}$, 1 mM EDTA and 1 mM MV^{2+} in B&R buffer pH 6, in the absence and presence of 16 µM LAC3. Same data as Figure 5 of the main paper but best fit (black solid lines) according to a simple reaction scheme considering parallel reduction of LAC3 by either $O_2^{\bullet-}$ or $MV^{\bullet+}$. (for fit parameters see Kinetic Simulation section below). Dashed green line: simulation of O_2 consumption in presence of LAC3 but without considering reduction of LAC3 by $MV^{\bullet+}$ ($k_{B5\text{-}B8} = 0$) keeping all other parameters constant. Comparing the dashed lines with the solid blue line indicates that $O_2^{\bullet-}$, and not $MV^{\bullet+}$, is the predominant reductant for the laccase under these conditions.

In the absence of laccase, the constant slope is determined by the dismutation of superoxide which is faster than its production (the steady state concentration of O_2^- is very low, see Fig. S13). In the presence of laccase, the initial O_2 consumption rate is zero because oxidation of O_2^- (regenerating O_2, no net consumption) is faster than dismutation. In other words, laccase prevents accumulation of sufficient concentration of O_2^- for dismutation to become kinetically competitive. Once LAC3 has recovered four electrons, the enzyme starts to consume O_2 by its normal catalytic activity. The action of O_2^- as a reversible electron relay between $MV^{\bullet+}$ and LAC3 is not contributing to O_2 consumption. Related to Fig. 5.

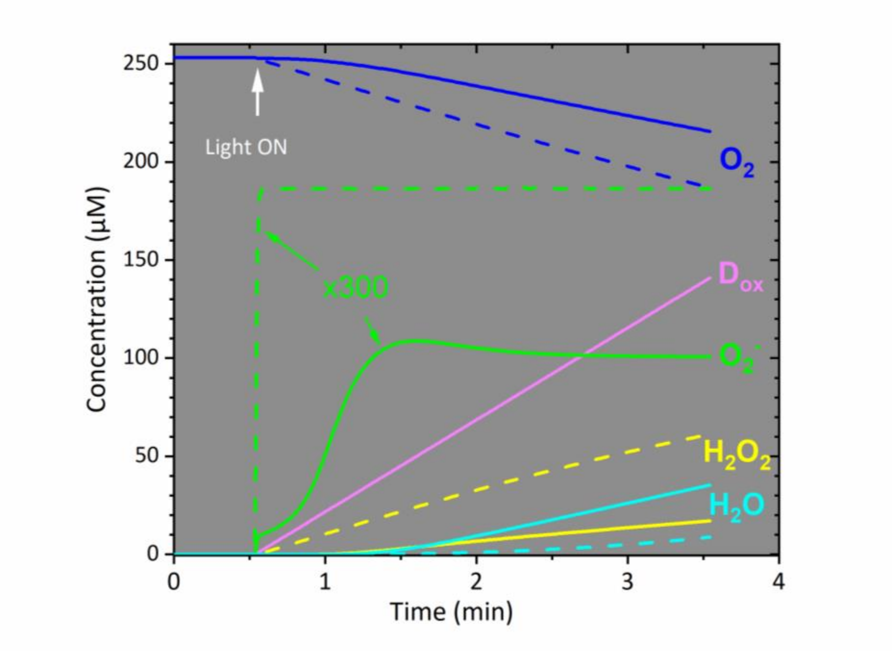

Figure S13. Visualization of the evolution of the concentration of key species during photocatalytic oxygen consumption measurements in the absence (dashed lines) and in the presence of 16 µM LAC3. D_{ox} is the concentration of oxidized electron donor. In presence of LAC3 production of H_2O_2 is strongly reduced (yellow traces) and production of H_2O is strongly accelerated. In the absence of LAC3 $O_2^{\bullet-}$ quickly reaches a low stead-state concentration (0.6 µM) given by the ratio of the constant rate of photoproduction via Ru* and $MV^{\bullet+}$ and the effective rate of dismutation which increases quadratically with $[O_2^{\bullet-}]$. When LAC3 is present $O_2^{\bullet-}$ level rises slowly because $O_2^{\bullet-}$ is initially efficiently oxidized by LAC3 until a steady-state level is established which is controlled by an ET step within the laccase which is rate limiting for O_2 reduction catalysis by laccase. Related to Fig. 5, Fig. S12, and Scheme 2.

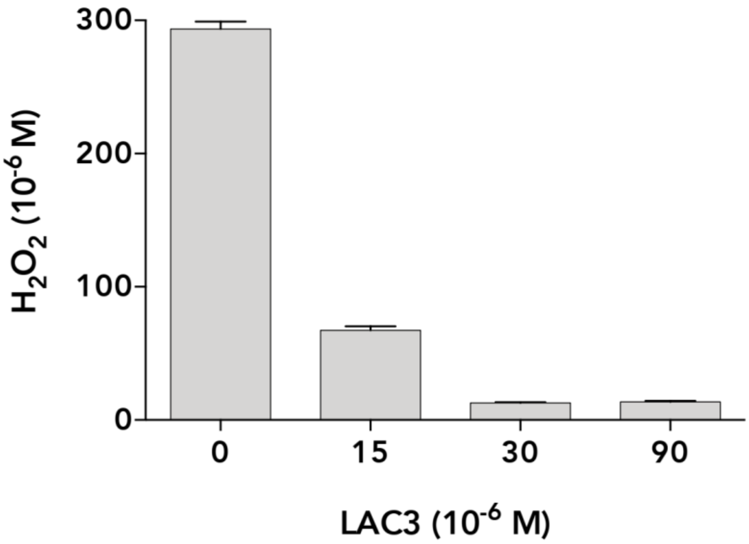

Figure S14. Light dependent H_2O_2 production as a function of LAC concentration. Conditions: in 1 mL Britton and Robinson buffer pH 4.0 (25°C) with [Ru]= 30 µM, [LAC3] = 0 to 90 µM, [EDTA] = 10 mM, and $[MV^{2+}]$ = 1.0 mM. Irradiation with a white Dolan-Jenner mi-LED (250 $mW.cm^{-2}$, $450<\lambda< 750$ nm) until 100 % O_2 consumed. Related to Fig. 5, Fig. S13, and Scheme 2.

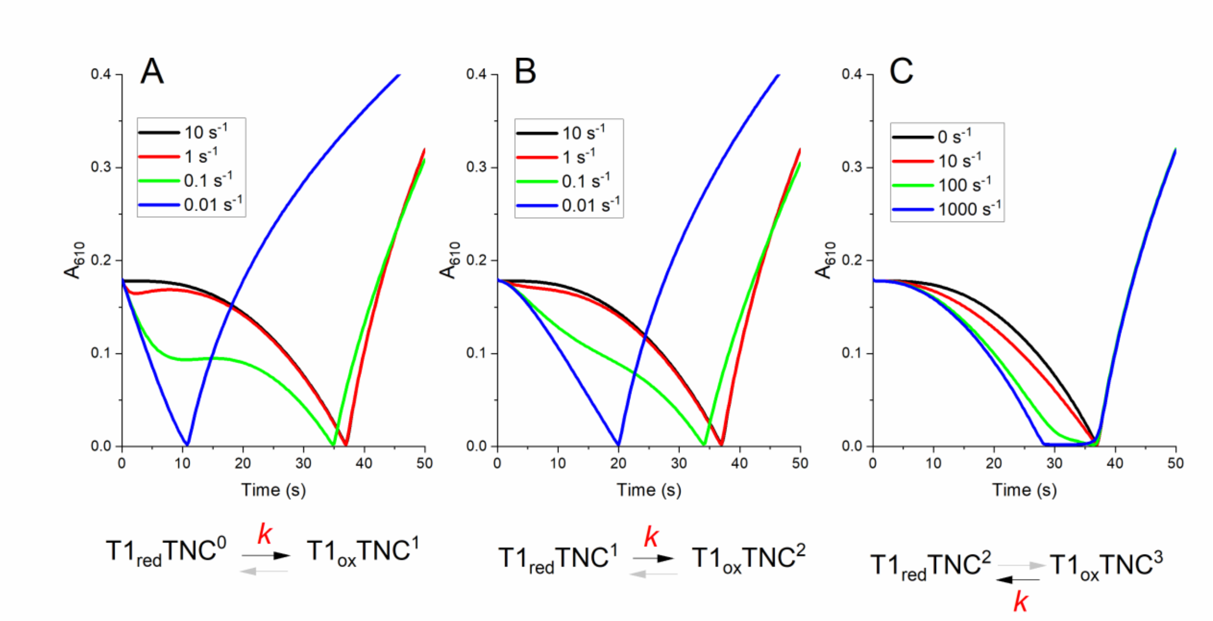

Fig. S15. Simulation of a typical anaerobic photoreduction experiment to visualize the sensitivity of the absorption changes at 610 nm on rate constants for internal electron transfer from T1 to the TNC. Presence of excess MV^{2+} (2 mM) is assumed which permits to neglect direct, inefficient interaction of the chromophore with laccase (reactions C1-C6 above). Light intensity was chosen to produce 3 µM/s of $MV^{\bullet+}$ reductant. A) Variation of the rate of the first IET from T1 to TNC; B) Variation of the rate of the second IET from T1 to TNC; C) Variation of the rate for reverse IET from the fully reduced TNC to T1. The latter corresponds to a change from an exergonic to an endergonic reduction step for the storage of the third electron on the TNC. All forward rate constants besides the ones varied are assumed not limiting (10 s^{-1}). All backward rate constants besides the one varied in panel C are set to 0. Related to Fig. 3.

Transparent Methods

Materials

Tris(2,2'-bipyridyl)dichlororuthenium(II) hexahydrate (99.5%), methyl viologen dichloride hydrate (98%) and benzyl viologen dichloride (97%) were purchased from Sigma-Aldrich. The laccase LAC3 from *Trametes* sp. C30 was produced as a recombinant enzyme in *Aspergillus niger* as described.(Klonowska et al., 2005, Mekmouche et al., 2014)

Laser flash photolysis

For transient absorption and emission measurements an Edinburgh Instruments LP920 flash photolysis spectrometer system was used with a Continuum Surelite OPO laser (5 ns pulse duration, typical energy 10 mJ) for excitation at 460 nm, a pulsed 450 W Xenon lamp for the probe light, and either a PMT (Hamamatsu) or ICCD camera (Andor) as detectors. Presented data are typically averages over 10-20 measurements. Samples were prepared in sealed quartz cuvettes containing Britton–Robinson (B&R) buffer at the indicated pH and were purged for 20 min with argon prior to each experiment.

Light-induced dioxygen consumption studies

Dioxygen consumption was measured by polarography using a model 781 oxygen meter (Strathkelvin Instruments) or a Hansatech Oxygraph system with a micro-Clark electrode fitted to a temperature-controlled glass chamber. Irradiation of the sample was performed through the glass chamber with a Dolan JENNER LED (250 mW.cm^{-2}) equipped with filters (450 nm $<\lambda<$700 nm). For the data in Fig. S11 irradiation was done with a Flexilux 600 Longlife 150 W 21 V halogen lamp through an ultraviolet cut-off ($\lambda \geq 375$ nm) and a 23% neutral density filter.

H_2O_2 quantification

H_2O_2 production after illumination as a function of laccase concentration was determined by HRP assay. 40 µL of the reaction solution, filtered through a 3 kDa filter was added to a solution of 240 µL ABTS (2 mg/mL) in phosphate buffer pH 8.0 and 10 µL HRP (Sigma, 0.103 mg/mL) in 100 mM phosphate buffer pH 8 was added. After 5 minutes of incubation the absorbance at 420 nm was recorded. Calibration was done with reference measurements in the absence of light.

Kinetic spectroscopic studies

UV-vis absorption spectra were recorded on a Cary 50 spectrophotometer equipped with a homemade irradiation setup including a Dolan JENNER LED (250 mW.cm^{-2}) equipped with filters (450 nm $<\lambda<$700 nm). For each MV^{2+} concentration a quartz cuvette was filled with the reaction mixture and sealed in a glovebox prior recording the corresponding spectrum.

Kinetic Simulation

Analysis of the kinetics of LAC3 photoreduction was performed using the KinTek Explorer simulation program (Johnson et al., 2009b, Johnson et al., 2009a).

A) Simulation of flash-induced absorption changes in absence of MV^{2+} (Fig. 1, Fig. S3)

The simulation is based on the reaction scheme shown in Scheme 1:

A1)	$Ru_{free} + L_{ox} \leftrightarrows RuL_{ox}$	formation of association complex
A2)	RuL_{ox} + light $\rightarrow Ru^{*}L_{ox}$	excitation in association complex
A3)	$Ru^{*}L_{ox} \rightarrow RuL_{ox}$	intrinsic excited state decay in complex
A4)	$Ru^{*}L_{ox} \rightarrow Ru^{III}L_{red}$	electron transfer
A5)	$Ru^{III}L_{red} \rightarrow RuL_{ox}$	back electron transfer
A6)	$Ru^{III}L_{red} \leftrightarrows Ru^{III} + L_{red}$	dissociation of charge separated state
A7)	Ru_{free} + light $\rightarrow Ru_{free}^{*}$	excitation of free chromophore
A8)	$Ru_{free}^{*} \rightarrow Ru_{free}$	excited state decay of free chromophore
A9)	$Ru_{free}^{*} + L_{ox} = Ru_{free} + L_{ox}$	excitation of free chromophore

The following rate constants were fixed: k_{A1}= k_{-A6} = $1{\cdot}10^9$ $M^{-1}s^{-1}$; k_{A2}= k_{A7} = $1{\cdot}10^9$ s^{-1}; k_{A8} = $1.7{\cdot}10^6$ s^{-1}. Extinction coefficients are given in the legend of Fig. 1. In the simulation excitation was induced after establishment of the association equilibria (reactions A1 and A6).

Best fit parameters: k_{-A1}= $2.1{\pm}0.045{\cdot}10^6$ s^{-1}; k_{A3}= $1.54{\pm}0.06{\cdot}10^6$ s^{-1}; k_{A4}= $8.48{\pm}0.3{\cdot}10^6$ s^{-1}; k_{A5}= $1.8{\pm}0.025{\cdot}10^6$ s^{-1}; k_{A6}= $0.12{\pm}0.002{\cdot}10^6$ s^{-1}; k_{A9}= $7.4{\pm}0.12{\cdot}10^6$ s^{-1}.

Association constants were determined as $K_a = k_{on}/k_{off} = k_{A1}/k_{-A1}$ and $K_a^{cs} = k_{-A6}/k_{A6}$.

B) Simulation of flash-induced absorption changes in presence of MV^{2+} (Fig. 2)

In presence of high concentrations of MV^{2+} ($[MV^{2+}] >> [Ru^{2+}]$) formation of association complexes between Ru^{2+} and LAC3 can be neglected. The simulation is based on the following reaction scheme starting with the flash-induced charge-separated state Ru^{3+} / $MV^{\bullet+}$ (initial concentrations 1.52 µM):

B1)	$Ru^{III} + MV^{\bullet+} \rightarrow Ru + MV^{2+}$	charge recombination
B2)	$MV^{\bullet+} + L_{ox} \rightarrow MV^{2+} + L_{red}$	LAC3 reduction
B3)	$Ru^{III} + L_{red} \rightarrow Ru + L_{ox}$	back electron transfer from reduced LAC3 to Ru^{3+}

Best fit parameters: k_{B1}= $3.1{\pm}0.01{\cdot}10^9$ $M^{-1}s^{-1}$; k_{B2} = $0.38{\pm}0.002{\cdot}10^9$ $M^{-1}s^{-1}$; k_{B3} = $0.46{\pm}0.004{\cdot}10^9$ $M^{-1}s^{-1}$.

When O_2 was present (Fig. S11) the following reaction steps were added:

B4)	$MV^{\bullet+} + O_2 \rightarrow MV + O_2^{\bullet-}$	oxidation of $MV^{\bullet+}$ radical by O_2
B5)	$Ru^{III} + O_2^{\bullet-} \rightarrow Ru + O2$	charge recombination between $Ru^{III} + O_2^{\bullet-}$

With k_{B4}= $0.7{\cdot}10^9$ $M^{-1}s^{-1}$; k_{B5} = $5{\cdot}10^9$ $M^{-1}s^{-1}$.

C) Simulation of anaerobic photoreduction of laccase (Fig. 3)

A simplified reaction scheme was used including the following reaction sequence:

C1)	Ru_L + light $\rightarrow Ru_L^*$	excitation in association complex
C2)	$Ru_L^* \rightarrow Ru_L$	intrinsic excited state decay
C3)	$Ru_L^* + T1_{ox}TNC^0 \rightarrow Ru^{III} + T1_{red}TNC^0$	
C4)	$Ru_L^* + T1_{ox}TNC^1 \rightarrow Ru^{III} + T1_{red}TNC^1$	effective reduction of laccase from
C5)	$Ru_L^* + T1_{ox}TNC^2 \rightarrow Ru^{III} + T1_{red}TNC^2$	the ruthenium excited state
C6)	$Ru_L^* + T1_{ox}TNC^3 \rightarrow Ru^{III} + T1_{red}TNC^3$	
C7)	$Ru^{III} + D \rightarrow Ru + D^+$	
C8)	Ru_{free} + light = Ru_{free}^*	excitation of free chromophore
C9)	$Ru_{free}^* = Ru_{free}$	intrinsic excited state decay
C10)	$Ru_{free}^* + MV^{2+} \rightarrow Ru^{III}_{free} + MV^{\bullet+}$	excitation and effective charge separation
C11)	$MV^{\bullet+} + T1_{ox}TNC^0 \rightarrow MV^{2+} + T1_{red}TNC^0$	
C12)	$MV^{\bullet+} + T1_{ox}TNC^1 \rightarrow MV^{2+} + T1_{red}TNC^1$	Laccase reduction by $MV^{\bullet+}$
C13)	$MV^{\bullet+} + T1_{ox}TNC^2 \rightarrow MV^{2+} + T1_{red}TNC^2$	
C14)	$MV^{\bullet+} + T1_{ox}TNC^3 \rightarrow MV^{2+} + T1_{red}TNC^3$	
C15)	$MV^{2+} + T1_{red}TNC^3 \leftrightarrows MV^{2+}{:}T1_{red}TNC^3$	interaction of reduced MV with fully reduced laccase
C16)	$Ru^{III} + MV^{\bullet+} \rightarrow Ru^{II} + MV^{2+}$	charge recombination
C17)	$T1_{red}TNC^0 \leftrightarrows T1_{ox}TNC^1$	
C18)	$T1_{red}TNC^1 \leftrightarrows T1_{ox}TNC^2$	internal ET from T1 to TNC
C19)	$T1_{red}TNC^2 \leftrightarrows T1_{ox}TNC^3$	
C20)	$Ru^{III}_{free} + D \rightarrow Ru_{free} + D^+$	oxidation of electron donor (EDTA)
C21)	$Ru_{free}^* + MV^{\bullet+} \rightarrow Ru_{free} + MV^{\bullet+}$	quenching of ruthenium excited state by energy transfer to $MV^{\bullet+}$

where:

- Ru, Ru*, and Ru^{III} stand for the ground state, triplet excited state, and oxidized state of the $[Ru(bpy)_3]^{2+}$ chromophore
- Ru_L is the fraction of chromophores in an association complex with laccase
- D and D^+ are the reduced and oxidized states of the sacrificial electron donor (EDTA)
- MV^{2+} and $MV^{\bullet+}$ are the oxidized and reduced states of the methyl viologen electron relay
- T1 and TNC are the type 1 Cu ion site, and the trinuclear Cu catalytic center in the LAC3 enzyme
- The LAC3 enzyme is described by its redox state from fully oxidized ($T1_{ox}TNC^0$) to the fully reduced form ($T1_{red}TNC^3$) with TNC^x, x = number of electrons on the TNC
- Reactions 3-6: the successive reductions of T1 by the excited state of the sensitizer in the preformed association complex
- Reactions 11-14: the successive reductions of T1 by $MV^{\bullet+}$
- Reaction 15 accounts for a hypothetical interaction of MV^{2+} with the fully reduced enzyme.
- Reactions 17-19: internal electron transfer reactions from T1 to the TNC in the laccase
- Reaction 21 accounts for energy transfer from Ru* to $MV^{\bullet+}$ having a broad absorption around 600 nm

Initial concentrations of chromophore-laccase association complexes (Ru_L) and free chromophore (Ru_{free}) were chosen according to the parameters determined before (Scheme 1). Other initial concentrations are those used in the experiment. Reaction 10 takes into account the escape yield (Sun et al., 1994). For computational reasons, effective electron transfer in association complexes between Ru and LAC3 were simulated by apparent rates for electron transfer (reactions 3-6) in competition with the other deactivation pathway for decay of the Ru excited state (reaction 2) and relative excitation rate (reaction 1). Potential charge recombination between Ru^{III}_{free} and $T1_{red}$ was neglected. All reactions except intramolecular ET in the laccase (17-19) and binding of MV^{2+} to fully reduced laccase (reaction 15) is considered irreversible. For some reactions, rate constants are known from independent laser flash kinetic measurements.

Fixed rate constants used: intrinsic excited state decay of Ru^*_{free}: k_{C9}= $1.6 \cdot 10^6$ s^{-1}; oxidation of EDTA by Ru^{III}: $k_{C7,C20}$ = $1.2 \cdot 10^6$ $M^{-1}s^{-1}$; energy transfer quenching of Ru^*_{free} by $MV^{\bullet+}$: k_{C21} = $8 \cdot 10^9$ $M^{-1}s^{-1}$. Forward rate constants for IET were fixed to $k_{C17-C19}$ = 1 s^{-1} (not limiting).

The essential result of the fit to the data is that, under the anaerobic conditions of this experiment where the fully oxidized form of the laccase enzyme corresponds to the resting oxidized form (Solomon, 2016), the third reduction of the TNC (reaction 19) is energetically uphill by about 200 meV. Values for rate constants for internal electron transfer in the laccase derived from the simulation: k_{-C17}=0.02 s^{-1}, k_{-C18}=0.85 s^{-1}, k_{-C19} ≈4500 s^{-1}.

Other fitted rate constants: k_{C10} = $0.3 \pm 0.19 \cdot 10^9$ $M^{-1}s^{-1}$, k_{C16} = $0.7 \pm 0.6 \cdot 10^9$ $M^{-1}s^{-1}$. Large standard errors like here indicate dependence between some of the parameters.

The traces in Fig. S10 were produced by plotting the flux integrals for reactions C7 and C20.

Simulations of a typical anaerobic photoreduction experiment to visualize the sensitivity of the absorption changes at 610 nm on rate constants for internal electron transfer from T1 to the TNC (reactions C17-C19) are shown in Fig. S15.

D) *Simulation of O_2 consumption (Fig. 5, Fig. S12, Fig. S13):*

We consider the following simplified reaction scheme with photoproduction of $MV^{\bullet+}$ via the Ru excited state as above, including charge recombination between Ru^{III} and $MV^{\bullet+}$ and reduction of Ru^{III} by the sacrificial electron donor EDTA. As under aerobic conditions all internal electron transfer steps are fast, we use the simplified representation of the five redox states of laccase L^0 - L^4 with L^0 the fully oxidized and L^4 the fully (4 e^-) reduced form. Protonation equilibrium of superoxide is assumed to be fast and is not explicitly considered.

D1)	Ru + light → Ru*	excitation and
D2)	$Ru^* + MV^{2+} \rightarrow Ru^{III} + MV^{\bullet+}$	effective charge separation
D3)	$Ru^{III} + D \rightarrow Ru^{II} + D^+$	oxidation of electron donor

D4)	$Ru^{III} + MV^{\bullet+} \rightarrow Ru^{II} + MV^{2+}$	charge recombination
D5)	$MV^{\bullet+} + L^0 \rightarrow MV^{2+} + L^1$	
D6)	$MV^{\bullet+} + L^1 \rightarrow MV^{2+} + L^2$	Laccase reduction by $MV^{\bullet+}$
D7)	$MV^{\bullet+} + L^2 \rightarrow MV^{2+} + L^3$	
D8)	$MV^{\bullet+} + L^3 \rightarrow MV^{2+} + L^4$	
D9)	$O_2^{\bullet-} + L^0 \rightarrow O_2 + L^1$	
D10)	$O_2^{\bullet-} + L^1 \rightarrow O_2 + L^2$	Laccase reduction by $O_2^{\bullet-}$
D11)	$O_2^{\bullet-} + L^2 \rightarrow O_2 + L^3$	
D12)	$O_2^{\bullet-} + L^3 \rightarrow O_2 + L^4$	
D13)	$L^4 + O_2 \rightarrow L^0 + 2\ H_2O$	4-electron reduction of O_2 by laccase
D14)	$MV^{\bullet+} + O_2 \rightarrow MV^{2+} + O_2^{\bullet-}$	O_2 reduction by $MV^{\bullet+}$
D15)	$O_2^{\bullet-} + O_2^{\bullet-} + 2\ H^+ \rightarrow H_2O_2 + O_2$	superoxide disproportionation
D16)	$H_2O_2 + H_2O_2 \rightarrow O_2 + 2\ H_2O$	H_2O_2 disproportionation

Initial concentrations were: [light] = 1 µM (constant), [Ru] = 30 µM, [D] = 1 mM, [MV^{2+}] = 1 mM, [L^0] = 0, 16 µM, [O_2] = 253 µM.

Fixed rate constants used: $k_{D2} = 0.3\cdot10^9\ M^{-1}s^{-1}$, $k_{D3} = 1.28\cdot10^6\ M^{-1}s^{-1}$, $k_{D4} = 1.27\cdot10^9\ M^{-1}s^{-1}$, $k_{D5\text{-}D8} = 0.5\cdot10^9\ M^{-1}s^{-1}$, $k_{D14} = 0.67\cdot10^9\ M^{-1}s^{-1}$, $k_{D15} = 1\cdot10^6\ M^{-1}s^{-1}$, $k_{D16} = 17\ M^{-1}s^{-1}$.

Rate constants determined by global fit of the O_2 consumption kinetics in Fig. 5:

$k_{D1} = 0.026\pm0.00002\ s^{-1}$, $k_{D9\text{-}D12} = 0.03\text{-}0.3\cdot10^6\ M^{-1}s^{-1}$, $k_{D13} = >10^3\ M^{-1}s^{-1}$,

Reaction 4 is essentially not operating due to the low steady-state concentrations of Ru^{III} and $MV^{\bullet+}$. At the high concentration of MV^{2+} used in the experiment laccase reduction directly from Ru* can be safely neglected.

Simulation of O_2 consumption with adduct (Fig. 5):

Same scheme as above with eqs D9-D12 replaced by:

D9a)	$O_2^{\bullet-} + L^0 \leftrightarrows O_2^{\bullet-}{:}L^0$	
D9b)	$O_2^{\bullet-}{:}L^0 \rightarrow O_2 + L^1$	
D10a)	$O_2^{\bullet-} + L^1 \leftrightarrows O_2^{\bullet-}{:}L^1$	
D10b)	$O_2^{\bullet-}{:}L^1 \rightarrow O_2 + L^2$	Laccase : $O_2^{\bullet-}$ adduct formation followed by
D11a)	$O_2^{\bullet-} + L^2 \leftrightarrows O_2^{\bullet-}{:}L^2$	laccase reduction
D11b)	$O_2^{\bullet-}{:}L^2 \rightarrow O_2 + L^3$	
D12a)	$O_2^{\bullet-} + L^3 \leftrightarrows O_2^{\bullet-}{:}L^3$	
D12b)	$O_2^{\bullet-}{:}L^3 \rightarrow O_2 + L^4$	

Fixed rate constants used: $k_{D9a\text{-}12a} = 0.5\cdot10^9\ M^{-1}s^{-1}$.

Rate constants determined by global fit of the O_2 consumption kinetics in Fig. 5: $k_{\text{-D9a--D12a}} = 0.9\pm0.8\cdot10^6\ M^{-1}s^{-1}$, $k_{D9b} = 2.6\pm0.6\cdot10^6\ s^{-1}$, $k_{D10b} = 0.38\pm0.05\cdot10^6\ s^{-1}$, $k_{D11b} = 0.08\pm0.08\cdot10^6\ s^{-1}$, $k_{D12b} = 0.23\pm0.5\cdot10^6\ s^{-1}$.

Other rate constants as before.

Calculation of energy and electron transfer rates

Förster-type resonance energy transfer:

To investigate the competition between energy transfer quenching and photo-induced electro transfer quenching of the ruthenium excited state it is useful to simulate the distance-dependence of both processes. The rate of oscillating dipole energy transfer between a donor and acceptor molecule can be deduced from equation (1) proposed by Förster (Főrster, 1959, Lakowicz, 2006)

$$k_{EnT}(r) = \frac{1}{\tau_D}\left(\frac{R_0}{r}\right)^6 \quad (1)$$

where τ_D the excited state lifetime of the donor in the absence of acceptor, R_0 is the Förster distance at which energy transfer is 50% efficient, and r is the distance between donor and acceptor. The coupling between the dipolar transitions of donor and acceptor is determined by the Förster radius given by

$$R_0^6 = \frac{9000*\ln(10)*\kappa^2*\Phi_D*J}{128*\pi^5*n^4*N_A} \quad (2)$$

where κ^2 is the dipole orientation factor, Φ_D the quantum yield of the donor emission, n the refractive index, N_A Avogadro's number, and J the spectral overlap integral

$$J(\nu) = \int_0^\infty F_D(\nu) * \varepsilon_A(\nu) * \nu^{-4}\, d\nu \quad (3)$$

in units of $mol^{-1}\ dm^3\ cm^3$ (Braslavsky et al., 2008), which contains the normalized emission spectrum of the donor, F_D, and the absorption spectrum of the acceptor, ε_A as a function of wavenumber, v. J was evaluated from the data in Fig. S1 to yield $J = 7.4 \cdot 10^{-14}\ mol^{-1}\ dm^3\ cm^3$. Using as values for the other parameters in eq (2) $\kappa^2 = 0.476$ (Steinberg, 1971), $\Phi_D = 0.042$ (Kalyanasundaram, 1982), and $n = 1.4$ Lakowicz (2006) R_0 is determined to $R_0 = 26.4$ Å. Together with the value $\tau_D = 588$ ns from Fig. S2 this allows to draw the $k_{EnT}(r)$ plot shown in Fig. S5.

Intra-complex electron transfer rate

Classical Marcus theory describes the rate of an exergonic reaction as (Marcus and Sutin, 1985, Millett and Durham, 2002)

$$k_{et}(\lambda, \Delta G°', r) = 1 * \ 10^{13} \exp(-\beta(r - r_0)) * \ \exp\left(-\frac{(\Delta G°' + \lambda)^2}{4\lambda k_B\, T}\right) \quad (4)$$

Where $\Delta G°'$ is the free energy of reaction, λ is the reorganization energy, r is the edge to edge distance between the two redox centers, r_0=3.6 Å is the van der Waals contact distance, and β =1.4 $Å^{-1}$. (Millett and Durham, 2002).

Supplemental References

BRASLAVSKY, S. E., FRON, E., RODRIGUEZ, H. B., ROMAN, E. S., SCHOLES, G. D., SCHWEITZER, G., VALEUR, B. & WIRZ, J. 2008. Pitfalls and limitations in the practical use of Forster's theory of resonance energy transfer. *Photochem Photobiol Sci,* 7**,** 1444-8.

FŐRSTER, T. 1959. 10th Spiers Memorial Lecture. Transfer mechanisms of electronic excitation. *Discussions of the Faraday Society,* 27**,** 7-17.

JOHNSON, K. A., SIMPSON, Z. B. & BLOM, T. 2009a. FitSpace explorer: an algorithm to evaluate multidimensional parameter space in fitting kinetic data. *Anal Biochem,* 387**,** 30-41.

JOHNSON, K. A., SIMPSON, Z. B. & BLOM, T. 2009b. Global kinetic explorer: a new computer program for dynamic simulation and fitting of kinetic data. *Anal Biochem,* 387**,** 20-9.

KALYANASUNDARAM, K. 1982. *Photophysics, photochemistry and solar energy conversion with tris(bipyridyl)ruthenium(II) and its analogues,* Amsterdam.

KLONOWSKA, A., GAUDIN, C., ASSO, M., FOURNEL, A., RÉGLIER, M. & TRON, T. 2005. LAC3, a new low redox potential laccase from Trametes sp. strain C30 obtained as a recombinant protein in yeast. *Enzyme and Microbial Technology,* 36**,** 34-41.

LAKOWICZ, J. R. 2006. Energy Transfer. *In:* LAKOWICZ, J. R. (ed.) *Principles of Fluorescence Spectroscopy.* Boston, MA: Springer US.

MARCUS, R. A. & SUTIN, N. 1985. Electron transfers in chemistry and biology. *Biochimica et Biophysica Acta (BBA) - Reviews on Bioenergetics,* 811**,** 265-322.

MEKMOUCHE, Y., ZHOU, S., CUSANO, A. M., RECORD, E., LOMASCOLO, A., ROBERT, V., SIMAAN, A. J., ROUSSELOT-PAILLEY, P., ULLAH, S., CHASPOUL, F. & TRON, T. 2014. Gram-scale production of

a basidiomycetous laccase in Aspergillus niger. *Journal of Bioscience and Bioengineering,* 117**,** 25-27.
MILLETT, F. & DURHAM, B. 2002. Design of photoactive ruthenium complexes to study interprotein electron transfer. *Biochemistry,* 41**,** 11315-24.
SOLOMON, E. I. 2016. Dioxygen Binding, Activation, and Reduction to H2O by Cu Enzymes. *Inorg Chem,* 55**,** 6364-75.
STEINBERG, I. Z. 1971. Long-range nonradiative transfer of electronic excitation energy in proteins and polypeptides. *Annu Rev Biochem,* 40**,** 83-114.
SUN, H., YOSHIMURA, A. & HOFFMAN, M. Z. 1994. Oxidative Quenching of the Excited-State of Tris(2,2'-Bipyridine)Ruthenium(2+) Ion by Methylviologen - Variation of Solution Medium and Temperature. *Journal of Physical Chemistry,* 98**,** 5058-5064.
WILSON, G. J., LAUNIKONIS, A., SASSE, W. H. F. & MAU, A. W. H. 1998. Chromophore-Specific Quenching of Ruthenium Trisbipyridine−Arene Bichromophores by Methyl Viologen. *The Journal of Physical Chemistry A,* 102**,** 5150-5156.
